# Hemocyanins of Muricidae: New ‘Insights’ Unravel an Additional Highly Hydrophilic 800 kDa Mass Within the Molecule

**DOI:** 10.1007/s00239-020-09986-6

**Published:** 2021-01-13

**Authors:** Gabriela Giannina Schäfer, Lukas Jörg Grebe, Frank Depoix, Bernhard Lieb

**Affiliations:** Institute of Molecular Physiology, Johannes Gutenberg-University of Mainz, Johann-Joachim-Becher-Weg 7, 55128 Mainz, Germany

**Keywords:** Hemocyanins, Muricidae, Gastropoda, Histidines, Protein evolution, Disulfide bridge

## Abstract

Hemocyanins are giant oxygen transport proteins that freely float within the hemolymph of most molluscs. The basic quaternary structure of molluscan hemocyanins is a cylindrical decamer with a diameter of 35 nm which is built of 400 kDa subunits. Previously published results, however, showed that one out of two hemocyanin subunits of *Rapana venosa* encompasses two polypeptides, one 300 kDa and one 100 kDa polypeptide which aggregate to typical 4 MDa and 8 MDa hemocyanin (di-)decamer molecules. It was shown that the polypeptides are bound most probably by one or more cysteine disulfide bridges but it remained open if these polypeptides were coded by one or two genes. Our here presented results clearly showed that both polypeptides are coded by one gene only and that this phenomenon can also be found in the gastropod *Nucella lapillus.* Thus, it can be defined as clade-specific for Muricidae, a group of the very diverse Caenogastropoda. In addition, we discovered a further deviation of this hemocyanin subunit within both species, namely a region of 340 mainly hydrophilic amino acids (especially histidines and aspartic acids) which have not been identified in any other molluscan hemocyanin, yet. Our results indicate that, within the quaternary structure, these additional amino acids most probably protrude within the inner part of didecamer cylinders, forming a large extra mass of up to 800 kDa. They presumably influence the structure of the protein and may affect the functionality. Thus, these findings reveal further insights into the evolution and structures of gastropod hemocyanins.

**Supplementary Information:**

The online version of this article (10.1007/s00239-020-09986-6) contains supplementary material, which is available to authorized users.

## Introduction

**Fig. 1:**
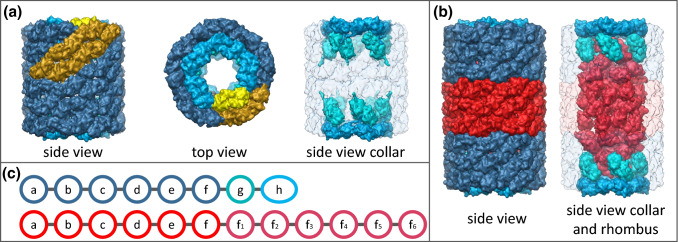
Gastropod hemocyanin didecamer and mega-hemocyanin tridecamer. **a** A typical gastropod hemocyanin didecamer is shown with a dark blue wall (FU-a–FU-f) and a cyan (FU-g) and light blue (FU-h) collar. It is based on the 9 Å model of KLH_1_ (Gatsogiannis and Markl 2009, PDB: 4BED). In the side and top view, one hemocyanin subunit dimer, which represents the repetitive unit within a molluscan hemocyanin decamer, is highlighted in golden with a yellow collar. In the side view of the collar, the wall is transparent to reveal the positions of FU-g and FU-h. **b** Mega-hemocyanin based on 3D volumes simulated by J. Markl in CHIMERA at 7 Å resolution on the basis of the pseudoatomic model presented in Gatsogiannis et al. (2015). The mega-hemocyanin comprises two typical hemocyanin decamers colored as described for (**a**). Between them, the 550 kDa subunit decamer is shown in red. In the right model, the wall is transparent to reveal the rhombus and the peripheral collar complex. **c** Scheme of a typical 400 kDa gastropod hemocyanin subunit (blue) and a 550 kDa mega-hemocyanin subunit build from multiple FUs (FU-h is about 100 amino acids larger). Color coding as described in **a** and **b**

Hemocyanins are blue, copper-containing oxygen transporters that freely float in the hemolymph of many arthropods and molluscs and, thus, are central proteins of the physiology of most species of the two largest animal phyla. Arthropod and molluscan hemocyanins are both very large multimeric proteins that share the same binuclear copper active site (van Holde and Miller 1995). Their primary, tertiary, and quaternary structures, on the other hand, are completely different. Burmester (2001) showed that they represent two distinct protein superfamilies. Since our work is focused on molluscan hemocyanins solely, we concentrate on the characteristics of the latter one. Molluscan hemocyanins form partly hollow cylinders consisting of decamers that can aggregate to didecamers or multidecamers (van Holde and Miller 1995; Markl 2013). The cylinders consist of a wall and an inner collar which is located typically at one end of the cylinder (Fig. 1a). With a diameter of 35 nm and a height of ≥ 18 nm, these large proteins can easily be seen in the transmission electron microscope. Hemocyanins also exhibit enormously large primary structures of up to 550 kDa (Lieb et al. 2010; Gatsogiannis et al. 2015). Each subunit covers several paralogous domains called functional units (FUs) which contain one oxygen-binding site each. The basic gastropod hemocyanin subunit consists of eight of these domains termed FU-a to FU-h, forming a polypeptide subunit of ca. 400 kDa (Fig. 1c). Each FU comprises about 420 amino acids with exception of the C-terminal FU-h that contains an additional tail of about 100 amino acids (van Holde and Miller 1995; Markl 2013).

In 2010, Lieb et al. discovered a gastropod hemocyanin that largely varies from the basic structure described above. It could be found exclusively within freshwater cerithioid snails and comprises two decamers formed by canonical 400 kDa hemocyanin subunits and additionally a central decamer consisting of 550 kDa subunits (Fig. 1c). In contrast to the typical partly hollow cylinder, this so-called mega-hemocyanin forms a cylinder which is almost completely filled (Fig. 1b). As the gastropod hemocyanin archetype, the 400 kDa subunit of this mega-hemocyanin tridecamer comprises the eight functional units FU-a to FU-h (Lieb et al. 2010). Thereby, FU-a to FU-f form the canonical wall of the cylinder while FU-g and FU-h build an inner collar. The 550 kDa subunit, however, is lacking FU-g and FU-h but covers, additionally to the characteristic FU-a to FU-f, six further functional units which are paralogous to FU-f and thus are termed FU-f_1_ to FU-f_6_ (Fig. 1c). They increase the inner collar which is normally built from FU-g and FU-h from ~ 100 to ~ 300 kDa (Lieb et al. 2010; Gatsogiannis et al. 2015). The resulting rhombus rises into the hollow parts of the flanking 400 kDa subunits and fills the tridecamer cylinder.

While these mega-hemocyanins do not exceed the outer dimension of other hemocyanin tridecamers consisting of three 400 kDa subunits, they carry 40 additional oxygen-binding sites. This increases the efficiency of oxygen transport without raising the viscosity or the colloid-osmotic pressure of the hemolymph (Gatsogiannis et al. 2015). Lieb et al. (2010) hypothesized that this may boost adaptive radiation of cerithioid snails, because mega-hemocyanins can evolve into high affinity as well as medium or low affinity forms which help to adapt to different habitats that could otherwise not be invaded (e.g., *Terebralia palustris* lives in hypoxic mangrove mud). In addition to these mega-hemocyanin tridecamers, typical decamers as well as di- and multidecamers that are comprised of exclusively 400 kDa subunits can be found in the hemolymph of Cerithioidea. Lieb et al. (2010) hypothesized that differential expression of the 400 kDa and 550 kDa subunits may help to respiratory acclimatize to different conditions.

Cerithioidea belong to Caenogastropoda, a group of very diverse gastropods living in all kinds of habitats. They have conquered land and freshwater several times independently and therefore have undergone a multitude of drastic evolutionary adaptations (Ponder et al. 2008). Another caenogastropod hemocyanin that has been intensely analyzed, so far, comes from *Rapana venosa* (e.g., Idakieva et al. 1993, 2012; Dolashka et al. 1996; Gebauer et al. 1999; Cheng et al. 2006). This species belongs to Muricidae, a clade of Neogastropoda which represent a sister group of Cerithioidea.

Gebauer et al. (1999) isolated two types of hemocyanin from the hemolymph of *R. venosa*, precisely RtH1 and RtH2 (named after the synonym *Rapana thomasiana*). Both paralogous hemocyanins represent archetypical 400 kDa subunits encompassing the functional units FU-a to FU-h (Gebauer et al. 1999) and constitute didecamers that form characteristic hemocyanin cylinders (Georgieva et al. 2005; Cheng et al. 2006). Gebauer et al. (1999) showed that RtH1 results in one 400 kDa band by SDS-PAGE as other typical gastropod hemocyanins. Biochemical analyses of RtH2, on the other hand, showed that RtH2 splits into two fragments under reducing conditions. They appear as two protein bands on SDS gels, one representing a 280 kDa fragment that covers FU-a–FU-f (RtH2.1), the other one a 120 kDa fragment constituting FU-g and FU-h (RtH2.2). In the absence of reducing agents, however, only one 400 kDa protein band can be seen for both hemocyanins. These results suggest the presence of one or more disulfide bridges linking both the wall-constituting FU-a to FU-f polypeptide and the collar-building FU-g/h fragment. Whether these two polypeptides arise due to a post-translational cleavage of the peptide or due to two different genes coding for two separate short subunits remained unclear until now (Gebauer et al. 1999). The evolution of a gene without FU-g and FU-h, however, could indicate a process similar to that which took place during the evolution of the mega-hemocyanin within the sister group Cerithioidea. This gene probably first lost FU-g and FU-h before it gained the additional domains through duplications of FU-f (Gatsogiannis et al. 2015).

To figure out the reason for the two hemocyanin fragments of RtH2, the intent of this study was to analyze the mRNA sequences of both hemocyanins of *Rapana venosa* and to check whether RtH2 is encoded by one or two genes. In addition, this study should reveal whether the separation of RtH2 into two fragments is a species- or genus-specific exception or a widespread phenomenon within the diverse family of Muricidae. Therefore, we additionally investigated the hemocyanins of *Nucella lapillus* by transmission electron microscopy (TEM), 3D reconstruction, SDS-PAGEs, and sequence analyses. In contrast to the marine *R. venosa,* this species of Muricidae lives intertidal and belongs to a different subfamily of Muricidae (Ocenebrinae instead of Rapaninae).

## Methods

### Animal Sampling and Hemocyanin Purification

Three individuals of *Nucella lapillus* were caught near to Morgat at the western Atlantic coast of Brittany, France, and stored in sea water. One individual of *Lymnaea stagnalis* was collected in a pond in Mainz, Germany. The hemolymph of one individual each was collected by cutting the foot muscle after the animals were kept on ice for 30 min. Pefabloc® SC was used as protease inhibitor (1 mM). Blood cells were removed by centrifugation at 800×*g* and 4 °C for 30 min. Hemocyanin was pelleted by ultracentrifugation at 4 °C for 2 h. The pellet was resuspended in a stabilizing buffer (50 mM Tris–HCl, 150 mM NaCl, 5 mM MgCl_2_, 5 mM CaCl_2_, pH 7.4) and stored at 4 °C.

### RNA and DNA Extraction and Next-Generation Sequencing

One individual of *N. lapillus* was sacrificed to isolate DNA from tissue of the foot using E.Z.N.A.® Mollusc DNA Kit (Omega Bio-Tek, Norcross, GA, USA). For RNA extraction mixed tissues including foot, hepatopancreas and mantle were prepared. RNA was isolated applying E.Z.N.A.® Total RNA Kit I (Omega Bio-Tek). Both DNA and RNA were purity checked and quantified via Nanodrop (Thermo Fisher Scientific, Waltham, MA, USA) and sent to StarSeq (Mainz, Germany) for next-generation sequencing (NGS, Illumina Next Seq500) and library preparation.

### In Silico Assembly of Hemocyanin Subunits

For hemocyanins of *N. lapillus*, transcriptomic NGS data which were sequenced as described above were used to assemble hemocyanin subunits. For those of *R. venosa,* we have used publicly available transcriptomic data (Acc. SRR2086477). Bioinformatic sequence analyses were performed using Geneious 9.1.8 (Kearse et al. 2012). Paired-end reads were set, raw reads were quality trimmed, and sequencing adapters were removed. Transcriptomic reads were then mapped to the previously published 400 kDa hemocyanin cDNA sequence of *Melanoides tuberculata* (KC405575) with a nucleotide identity of 70%. Mapped reads were isolated and used as references for iteratively mapping of further transcriptomic reads to prolongate the various hemocyanin fragments. To assure an accurate assembly, highly sensitive mapping settings were used (minimum overlap: 60 nucleotides; minimum overlap identity: 99%; maximum mismatches: 1%). This procedure was reiterated until all gaps between these fragments were closed and hemocyanin fragments of both species could be assembled to full-length hemocyanin coding sequences. For *N. lapillus*, also genomic NGS data were used to fill sequence gaps and to confirm assemblies.

Since both species include two hemocyanins and additionally each hemocyanin cDNA includes eight paralogous functional unit domains with some highly conserved sequence sections, this method may incorrectly merge different hemocyanin cDNA sequences. This could lead to sequence hybrids. Therefore, it is of general importance for sequence assemblies of molluscan hemocyanins to check the resulting sequences carefully and to manually examine highly identical sequence sections to preclude wrong assemblies. In this study, we verified the resulted sequences by (i) additionally mapping the total dataset of reads to the resulted hemocyanin sequences with low sensitive mapping settings which allow misassembly detection (small number of required overlapping nucleotides, here 25 nucleotides, and high number of nucleotide mismatches allowed beyond the overlapping region, here 60% of the total read length), (ii) aligning the resulted sequences to check for highly identical sequence sections (for results see Supplement 1), and (iii) using paired-end reads for sequence assemblies which enabled us to check that repetitive sequences are spanned by paired-mates.

In addition to transcriptomic data, genomic NGS data (for *N. lapillus sequenced* by StarSeq, for *R. venosa* publicly available data: SRR5371534) were used to check the genome for further hemocyanin subunits by mapping them to the compiled hemocyanin sequences with low mapping identity.

### Sequence Alignment and Phylogenetic Tree

**Fig. 2:**
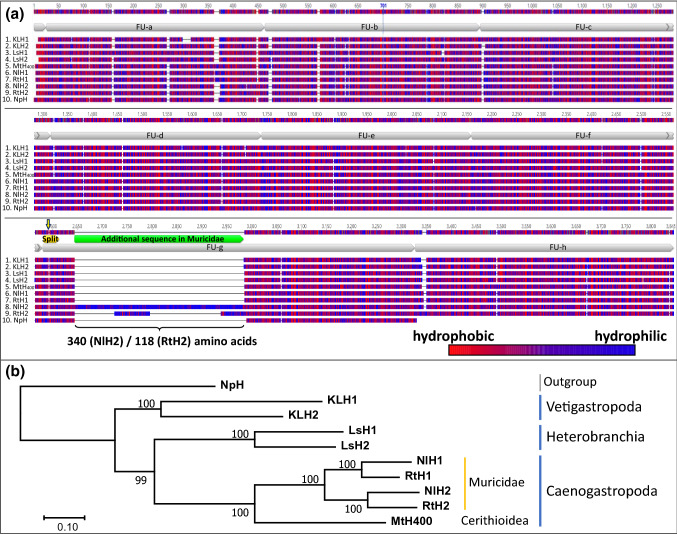
Phylogenetic sequence analysis. The sequence alignment (**a**) and the maximum likelihood-based phylogenetic tree (**b**) include nine gastropod hemocyanins and one hemocyanin of *Nautilus pompilius* (NpH) as a representative of a non-gastropod mollusc. The hemocyanin sequences included are from the vetigastropod *Megathura crenulata* (KLH, from keyhole limpet), the heterobranch *Lymnaea stagnalis* (LsH) and the caenogastropods *Melanoides tuberculata* (MtH_400_), *Nucella lapillus* (NlH), and *Rapana venosa* (RtH). NlH and RtH are derived from our study. **a** The alignment includes the full-length amino acid sequences of hemocyanins. Amino acids are color coded according to their hydrophobicity (red most hydrophobic; blue most hydrophilic). Their functional units are shown with gray bars below the consensus sequence (top line). The arrow above the yellow bar in FU-g marks the position in which RtH2 is split into two fragments according to Gebauer et al. (1999). The green bar highlights the additional highly hydrophilic sequence sections found in RtH2 and NlH2. The detailed alignment is presented in supplement 2. **b** The maximum likelihood tree is not intended to represent a full phylogeny but only reflecting the interrelation of the analyzed hemocyanins of Muricidae within the selected hemocyanins. It is based on the alignment in **a**, calculated with the WAG + G + F model and rooted with NpH (*Nautilus pompilius hemocyanin*)

Amino acid sequences were aligned by the MUSCLE algorithm implemented in MEGA version 7 (Kumar et al. 2016). Geneious 9.1.8 (Kearse et al. 2012) was used to display the alignment with the annotations depicted in Fig. 2. We used MEGA version 7 (Kumar et al. 2016) to determine WAG + G + F to be the best model of evolution for the alignment and to conduct the maximum likelihood analyses based on this model. Branch support was evaluated using 100 bootstrap replicates. Additionally to the hemocyanins of *N. lapillus* and *R. venosa*, these analyses include the deduced amino acid sequences of the following hemocyanin cDNAs to enable phylogenetic classification and the interrelation of NlH1 + NlH2 and RtH1 + RtH2: KLH1 and KLH2 (CAG28309.2, CAG28310.1, *Megathura crenulata* / keyhole limpet), LsH1 and LsH2 (AYO86691.1, AYO86692.1, *Lymnaea stagnalis*), MtH_400_ (AGX25261.1, *Melanoides tuberculata*), and NpH (CAF03590.1, *Nautilus pompilius*; used to root the phylogenetic tree).

### Biochemical Analysis

To dissociate hemocyanin into its subunits, freshly purified samples of NlH and LsH were dialyzed overnight against a 130 mM glycine/NaOH buffer at pH 9.6. Hemocyanin of *Rapana venosa* was used from the studies of Gebauer et al. (1999). SDS-PAGE was performed in a 10% polyacrylamide gel according to Laemmli (1970) using β-mercaptoethanol as reducing agent. One sample of NlH was prepared without β-mercaptoethanol. Protein bands were stained using colloidal Coomassie® brilliant blue R250 (Serva).

### Transmission Electron Microscopy (TEM) and Image Processing

Negative staining TEM was done as described previously by Harris (2007) using continuous carbon films and 1% uranyl acetate with a protein concentration of 0.1 mg/ml. A FEI Tecnai 12 (bioTwin) transmission electron microscope was used at an accelerating voltage of 120 kV. Images were taken with a 1392 × 1040 SIS Megaview camera. For the 3D reconstruction, images were automatically collected using the LEGINON system (Suloway et al. 2005) and processed using cisTEM (Grant et al. 2018; Zivanov et al. 2018). 9,800 particles were used from 271 micrographs.

## Results

In order to analyze the hemocyanins of *Rapana venosa* and *Nucella lapillus* more deeply, we sequenced and assembled transcriptomic NGS data to obtain their coding sequences and additionally analyzed hemocyanins we extracted from the hemolymph of *N. lapillus* by SDS-PAGEs and transmission electron microscopy (TEM).

### NGS Data Reveal an Atypical Hydrophilic Region Within Hemocyanin Subunits RtH2 and NlH2

Assembling the NGS data, we were able to construct two complete hemocyanin coding sequences of *Rapana venosa* (RtH1: BK014286; RtH2: BK014286) and two of *Nucella lapillus* (NlH1: MT939254; NlH2: MT939255). Details for each cDNA and the obtained primary structure are shown in Table 1. Assembled sequences were verified as described under *methods* section (detailed results on sequence comparison and confirmation in Supplement 1). The analysis revealed that the two hemocyanin polypeptides of both species are encoded by one gene each. Further paralogous hemocyanins were neither detected within transcriptomic nor in genomic data.

**Table 1:** Hemocyanins of *Rapana venosa* and *Nucella lapillus*

	RtH1	RtH2	NlH1	NlH2
Accession number	BK014286	BK014287	MT939254	MT939255
Length of coding sequence (bp)	10,320	10,659	10,323	11,307
Length of primary structure (aa)	3440	3553	3441	3769
Molecular weight (kDa)	394	409	396	437

Shown are lengths of coding sequences in base pairs (bp); number of amino acids (aa) for the deduced primary structure of the polypeptides and the calculated molecular weight in kDa

The alignment shown in Fig. 2a reveals that RtH2 and NlH2 encompass amino acids within the N-terminal region of FU-g which are additional to the highly conserved polypeptide structure of typical FU-gs or FUs in general (green bar in Fig. 2a). Compared to the sequences of other gastropod hemocyanin FUs, FU-g is extended by a sequence of 118 amino acids in RtH2 (13.6 kDa) and 340 amino acids in NlH2 (41.4 kDa), respectively. These sequences, which have not been observed in any other molluscan hemocyanin yet, are remarkably histidine and aspartic acid rich. Those two amino acids represent over 60% of the amino acids within that sequence section of RtH2 and over 70% within that of NlH2. Thus, in both polypeptides, this part of the sequence is extremely hydrophilic (color coding in Fig. 2a).

The phylogenetic analysis (Fig. 2b) reveals that RtH1 and NlH1 as well as RtH2 and NlH2 are orthologous genes. Thus, the duplication event which led to these two genes most probably took place in a precursor of both Muricidae species.

### Polypeptide Split of Hemocyanin Subunits is Muricidae-Specific

**Fig. 3:**
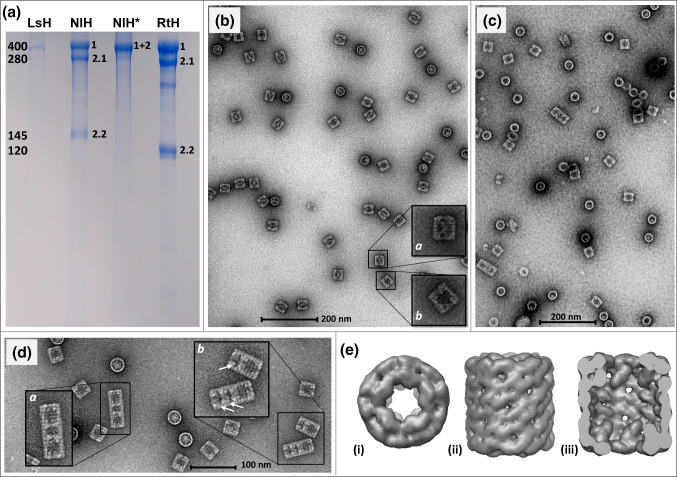
Analysis of SDS-PAGE, transmission electron microscopy (TEM), and 3D reconstruction of the hemocyanin of *Nucella lapillus*. **a** Coomassie-stained gel obtained by SDS-PAGE with and without (asterisk) β-mercaptoethanol. LsH: *Lymnaea stagnalis* hemocyanin (marker for 400 kDa subunit). NlH: *Nucella lapillus* hemocyanin. NlH*: NlH under non-reducing conditions. RtH: *Rapana venosa* hemocyanin. **b**–**d** TEM of negatively stained hemocyanin from *Nucella lapillus* (**b, d**) and *Megathura crenulata* (**c**). **b** The electron micrograph shows hemocyanin molecules of *N. lapillus* with two appearances of didecamers which may represent two different types of hemocyanin: One side view of each potential type is shown in subwindows on the right side in a higher magnification. Both types build cylinders of about 35 nm in diameter. While one appears partly hollow as typical for molluscan hemocyanins (*b*), the other regularly appearing type seems to be semi-filled (*a*) as it has not been observed before for molluscan hemocyanin didecamers. For comparison, a micrograph of hemocyanins of *M. crenulata* (KLH) is shown in **c**. For KLH, no didecamer side view appears to be semi-filled (compare also overview of negatively stained hemocyanins from different species in Markl (2013)). The electron micrograph in **d** depicts hemocyanin multidecamers of *N. lapillus* (higher magnification in the subwindows). The pentadecamer in **d**(*a*) comprises two typical didecamers (wall and inner collar on both ends) and additionally a monodecamer between them (*). This monodecamer (marked with the asterisk) appears to lack a gastropod hemocyanin characteristic inner collar. In comparison to that the tridecamer and tetradecamer in **d**(*b*) include—besides to one didecamer each—typical monodecamers which comprise an inner collar (marked with arrows). **e** 13 Å structure of NlH in top view (*i*), side view (*ii*), and cut-open side view (*iii*)

Total NlH was purified from the hemolymph of *N. lapillus* and dissociated into its subunits by dialysis. By SDS-PAGE analysis, we were able to identify three distinct protein bands in the Coomassie-stained gel (Fig. 3a, lane 2). For calibration, the known hemocyanins of *Lymnaea stagnalis* which represents typical 400 kDa hemocyanins were used as protein marker (lane 1). Furthermore, we included the hemocyanins of *R. venosa* in the analysis (lane 4) to enable comparisons with the results of Gebauer et al. (1999). Figure 3a shows that under reducing conditions, the sample of NlH includes one typical 400 kDa hemocyanin (NlH1), as well as one hemocyanin fragment of about 280–290 kDa as *R. venosa* does (NlH2.1). The third protein fragment runs similar but clearly above the 120 kDa RtH2.2 fragment of *R. venosa* (NlH2.2 = 145 kDa). As Gebauer et al. (1999) have already shown for RtH, the NlH sample without β-mercaptoethanol (NlH* in Fig. 3a) results in only one protein band of about 400 kDa. The gel additionally shows a fourth protein band for the hemocyanins of *R. venosa* which appears when *RtH* was stored for several months (Gebauer et al. 1999) and most probably contains degradation products (e.g., fragments of three FUs).

### Transmission Electron Microscopy Indicates an Extra Mass Within one Type of NlH Didecamers

The electron micrograph of the negatively stained total native NlH in Fig. 3b shows hemocyanin didecamer cylinders, as they are typical for molluscan hemocyanins. While some of the particles appear as canonical 400 kDa hemocyanins (partly hollow cylinders; Fig. 3b(*b*)), others seem to be semi-filled with an additional mass (semi-filled cylinders; Fig. 3b(*a*)). The comparison of the observed hemocyanin cylinders of *N. lapillus* with those of KLH (Fig. 3c) and transmission electron micrographs of other hemocyanins (e.g., overview in Markl (2013)) shows that the observed appearances most probably do not result from a typically hollow 400 kDa hemocyanin. Such semi-filled didecamers have not been detected for any other gastropod hemocyanin before.

In addition to didecamers, the hemocyanin of *N. lapillus* forms multidecamers. Examples are shown in the electron micrograph in Fig. 3d. The tri- and tetradecamer in Fig. 3d(*b*) include solely (di)decamers with an inner collar (marked with arrows) as typical for hemocyanins. In contrast to that, the pentadecamer in Fig. 3d(*a*) comprises a decamer which appears to lack an inner collar (marked with an asterisk).

To receive more information on the hypothetical additional mass inside of the hemocyanin didecamer cylinders, we rendered a 3D reconstruction of NlH. Using approximately 9,800 didecamers from negative stain TEM, we obtained one 13 Å model with a structure similar to that of KLH and without an additional mass in the center (Fig. 3e). Thereby, it was impossible to distinguish between the two potentially different types of molecules (filled and unfilled). In order to ensure that the proposed additional masses are not only misinterpreted views of the reconstructed unfilled molecule, we compared the images of TEM with back projections from the 3D model. The back projections clearly did not reveal any indications of the proposed extra mass within the center of the didecamer. Thus, we conclude that the reconstruction failed to resolve an inner mass. This may imply that if there is an additional mass, it will most likely be highly flexible within the center of the protein. It is a well-known phenomenon of single particle analyses not to resolve highly flexible parts of molecules because this method is founded on image averages of the same views of molecules. Structural flexibilities will be averaged out when merged to a 3D model (Orlova and Saibil 2010; Durand et al. 2013).

## Discussion

As many other molluscs, the marine gastropod *Rapana venosa* has two paralogous 400 kDa hemocyanins (Gebauer et al. 1999), both including the functional units FU-a to FU-h. In contrast to all other known molluscan hemocyanins, however, one of these hemocyanin subunits, precisely RtH2, consists of two polypeptides (RtH2.1 + RtH2.2) which most probably are bound by one or more disulfide bridges (Gebauer et al. 1999). Thus, our particular focus of attention was directed towards the second hemocyanin isoform of *Rapana venosa*. To find out if this peculiarity is species-specific, we additionally investigated hemocyanins of *Nucella lapillus*, another species of Muricidae which in contrast to *R. venosa* represents an intertidal living gastropod.

We were able to assemble the full-length coding sequences of two hemocyanins within both species and our analyses revealed that *N. lapillus* possesses two hemocyanins (NlH1 and NlH2) that are orthologous to RtH1 and RtH2. Biochemical investigations by SDS-PAGEs showed that NlH2 consists of two polypeptides, as well (Fig. 3a). NlH2.1 is similar in size as RtH2.1 (~ 280–290 kDa) and most probably comprises FU-a to FU-f. In contrast to that, NlH2.2 (~ 145 kDa) is about ~ 25 kDa larger than RtH2.2 (~ 120 kDa; FU-gh). The difference in size of NlH2.2 and RtH2.2 can be explained by the lengths of the amino acid sequences deduced from the cDNA. NlH2 is about 220 amino acids longer than RtH2 and has a calculated molecular weight which is about 28 kDa higher than that of RtH2 (Table 1). This corresponds to the difference between the protein bands of the gel (~ 25 kDa; Fig. 3a). The alignment, shown in Fig. 2a, reveals that the varying number of amino acids are located within FU-g and thus are part of RtH2.2 and NlH2.2, respectively. Additionally, our results proved that RtH2 and NlH2 are encoded by one gene each and that the amino acid chain must post-translationally be split into two polypeptides.

**Fig. 4:**
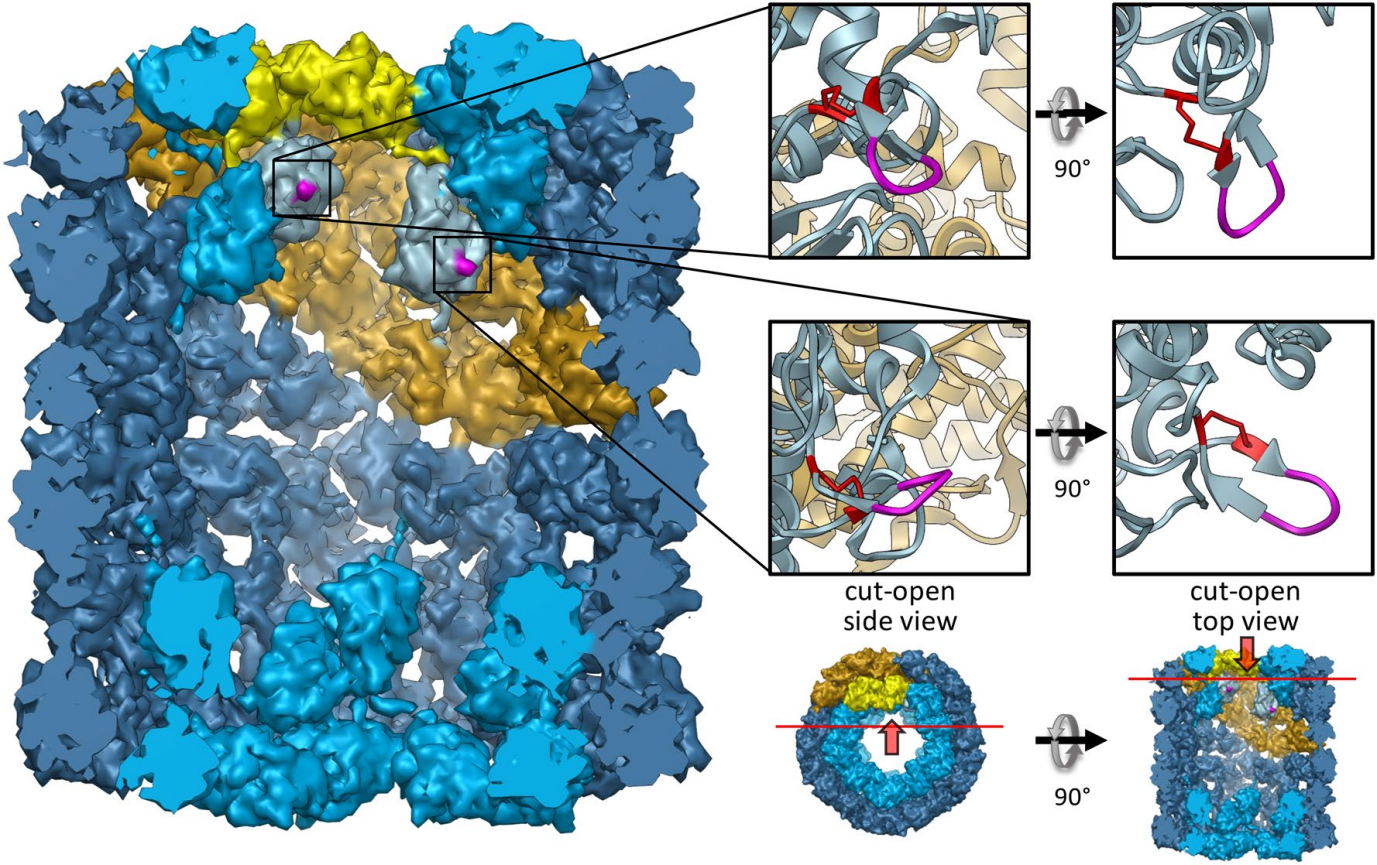
Location of the additional amino acids of RtH2 and NlH2 marked in a typical molluscan hemocyanin didecamer molecule. Shown is the model of a KLH1 didecamer based on a 9 Å cryoEM structure (Gatsogiannis and Markl 2009, PDB: 4BED). On the left side, the inner surface of the cylinder is shown by a cut-open side view (the cutting surface is represented by the red line in the top view model, next to it at the bottom). Color coding: dark blue: wall (FU-a–FU-f); light blue: inner collar (Fu-g and FU-h). One subunit dimer is highlighted in golden (wall) and yellow (collar) whereas FU-g of this dimer is distinctly marked in silver. The pink part of FU-g represents the position of the primary structure which is interrupted by the additional amino acids in RtH2 and NlH2. On the right, magnifications of the ribbon of this section are depicted. Left: cut-open side view. Right: cut-open top view. The smaller density models below illustrate the lines of sight (red arrows) and the cutting surfaces (red lines). The loop marked in pink is conserved in hemocyanins over all molluscan classes that have been analyzed so far and typically covers four to seven amino acids (five in KLH1). It is stabilized by a disulfide bridge marked in red. Within one hemocyanin isoform of both Muricidae species, however, it is interrupted by 118 (RtH2) and 340 (NlH2) amino acids, respectively. The folding of these additional amino acids and the resulting structure of the extra mass within the hemocyanin cylinder is unknown and, thus, not included in this figure

In comparison with other molluscan hemocyanins, both species we analyzed in this study include additional amino acids in their primary structure which are located within the N-terminal region of FU-g (Fig. 2a). These additional sequences encompass 118 amino acids in RtH2 and 340 amino acids in NlH2 which correspond to 13.6 kDa and 41.4 kDa, respectively. The hemocyanin didecamer model in Fig. 4 shows the location of the amino acids in FU-g within the KLH molecule where the primary structure of NlH2 and RtH2 indicates the insertion of additional amino acids (marked in pink). The depicted model shows the hemocyanin of the gastropod *Megathura crenulata* (KLH1). Nevertheless, the structures are well comparable because our electron microscopical results on NlH as well as the studies of Cheng et al. (2006) on RtH show that their structures of didecamers correspond to the basic composition of hemocyanin cylinders. This is highly conserved over all molluscan hemocyanins (cf. e.g., Orlova et al. 1997; Meissner et al. 2007; Gatsogiannis et al. 2007; Gatsogiannis and Markl 2009; Markl 2013; Gai et al. 2015). Since the amino acids which frame the extra polypeptide section in RtH2 and NlH2 are located at the inner surface of the didecamer cylinder, we hypothesize that the additional amino acids reach into the inner part of the molecule and form an extra mass within the center of the hemocyanin didecamer.

The strong polarity (Fig. 2a) of the additional sequence section of the polypeptide chain further indicates that the extra mass reaches into the cavity of the molecule because this facilitates an interaction with the solvent (Zhou and Pang 2018). Furthermore, we would expect that the highly hydrophilic sequence sections would most probably change the folding of the proteins significantly, if they reached into the cylinder wall or collar structure, because additional amino acids would influence hydrophobic interactions of nonpolar amino acid residues. These, however, are typically located within proteins and are eminently important to stabilize their folding (Schreiber et al. 2009; Nick Pace et al. 2014). Since the cylindrical structures observed for NlH and RtH are rather typical for hemocyanins (Fig. 3b, d; Cheng et al. 2006), this also supports the hypothesis that the additional amino acids reach within the center of the molecule.

By TEM, we identified two appearances of NlH didecamers. While one represents a typical didecamer, the other one potentially comprises an additional mass lying within its center. Negative staining may cause artifacts and thus these results have to be considered carefully. Comparing the results with other negative staining TEM results, however, such an inner mass has not been detected yet for any other hemocyanin and may also indicate that the additional amino acids reach within the center of the hemocyanin molecules. It has to be regarded that such an additional mass was not detected for the hemocyanins of *R. venosa* (Georgieva et al. 2005; Cheng et al. 2006). This could be traced back to the size of the additional mass, which is reduced for RtH2. The additional hydrophilic sequence section of NlH2 is 2.5 to 3 times larger than that of RtH2 and would aggregate to an extra mass encompassing 828 kDa within a total didecamer molecule of NlH2. This even corresponds to the size of two complete hemocyanin subunits. In a didecamer composed of RtH2, in contrast, the potential emerging additional mass would correspond to only 272 kDa which could easily be undetected by analyzing electron micrographs.

Although we were able to achieve a reconstruction with a resolution of 13 Å from negative stained electron micrographs of NlH, the additional mass (~ 828 kDa) could not be resolved in a 3D model (Fig. 3e). This suggests a highly variable structure, since it is a well-known aspect of single particle analysis that very flexible parts will be averaged out during image processing (Orlova and Saibil 2010; Durand et al. 2013). Such a high flexibility of the additional sequence section could result from its composition of repetitive amino acid motifs that encompass about 70% histidines and aspartic acids in NlH2. These amino acids can interact not only with the solvent (polarized bonds and ionization) but also with each other due to van der Waals interactions and *π*–*π* stacking of the aromatic systems (Trevino et al. 2007; Liao et al. 2013). Thereby, the large number of identical amino acids can facilitate a multitude of interactions between varying residues within one and between several subunits. This could result in different formations and a highly variable structure of the additional mass within the center of a hemocyanin didecamer cylinder and might therefore be an explanation why the additional mass cannot be resolved within a 3D reconstruction.

Despite these presumably flexible extra masses, the electron micrographs clearly show an inner collar in both types of molecules which are typical for gastropod hemocyanins (Fig. 3b, d; cf. Markl 2013). Also, the final model obtained by a 3D reconstruction includes a typical inner collar. Since it was not possible to distinguish between the two types of hemocyanins, the reconstruction indicates that—apart from the presumably highly flexible structures which are averaged out—the two potentially different molecules are very similar to each other. This substantiates the assumption that NlH1 and NlH2 both form an inner collar within the hemocyanin didecamers and therefore indicates that the two polypeptide fragments of NlH2 (FU-abcdef and FU-gh) are most probably congregated within the didecameric molecule.

Besides didecamers, we observed multidecamers of hemocyanins (Fig. 3d). Among them, we found multidecameric structures which incorporate decamers without an inner collar (asterisk in Fig. 3d). This suggests that these decamers are built by subunits consisting solely of FU-a to FU-f. Since we did not find any further hemocyanin genes, such decamers lacking an inner collar must descend from the genes of NlH1 or NlH2. One possible explanation for that phenomenon could be an alternative splicing. We, however, suggest that NlH2 could result in such decamers without an inner collar due to a post-translational loss of the protein fragment NlH2.2 which was detected by SDS-PAGEs (Fig. 3a) and presumably comprises FU-g and FU-h. Our results show that under non-reducing conditions, NlH2 does not split into two fragments as it does during SDS-PAGEs including β-mercaptoethanol (Fig. 3a). This suggests the presence of one or more inter-FU disulfide bridges between the two hemocyanin polypeptide chains NlH2.1 and NlH2.2 and confirms the results of Gebauer et al. (1999) which revealed the same phenomenon for RtH2. The non-appearance or loss of the hypothesized disulfide bridges in some cases could eventually cause decamers without inner collars. Subunits without FU-g and FU-h can form stable hemocyanin decamers as shown in *Biomphalaria glabrata*, a gastropod which uses multimeric hemoglobin as oxygen carrier but additionally still expresses “rudimentary” hemocyanins (Lieb et al. 2006).

All molluscan hemocyanins that have been analyzed so far have disulfide bridges within their functional units (Gielens et al. 1997; Cuff et al. 1998; Georgieva et al. 2004; Bergmann et al. 2007). An inter-FU disulfide bond as hypothesized by Gebauer et al. (1999) and additionally indicated by our here presented results, however, has not been proven so far. We have neither obtained an atomic structure of NlH2 or RtH2 nor is there any hemocyanin model with a comparable structure inside of the cylinder published so far which could be used to fit the sequence into a model. Thus, it is impossible to assert a disulfide bridge between different FUs. However, our sequence data show that hemocyanins of both analyzed species comprise a cysteine additionally to the highly conserved cysteines which typically build intra-FU disulfide bonds. This additional cysteine is located in the N-terminal region of FU-d (see position 1348 in alignment, Supplement 2) and has not been detected in any other molluscan hemocyanin, yet. The figure in Supplement 3 shows that the position where this cysteine evolved is located on the inner surface of the wall of the KLH1 didecamer cylinder. Thus, it might be accessible from the center of the molecule. One highly speculative scenario we propose is that this cysteine may potentially interact in a disulfide bond with one of the cysteines in FU-g which typically form the disulfide bridge that stabilizes the loop shown in Fig. 4 (marked in pink). In RtH2 and NlH2, these two cysteines are spatially separated within the primary structure from each other due to the incorporation of the additional amino acids (in the conserved hemocyanin amino acid sequence, only eight amino acids are located between them, while it would be 118 and 340 in RtH2 and NlH2, respectively; Supplement 2). This may, for example, impede the primordial disulfide bridge in quaternary structure and may enable another disulfide bond, e.g., with the described cysteines in FU-d. Mass spectroscopic analysis could help to reveal the structure of RtH2 and NlH2 and to provide further information on the role of the specific disulfide bridge marked in pink in Fig. 4, which most probably stabilizes the core domain including the oxygen-binding site of all functional units (Georgieva et al. 2004).

All the results of this study indicate a diversification of FU-g within one hemocyanin isoform of both Muricidae species, *Nucella lapillus* and *Rapana venosa*. Although we were not able to resolve the three-dimensional structure of the extra mass, the additional amino acids included in the primary structure inevitably must change the folding and structure of this functional unit. This may also be associated with a functional change or loss. Since the two cysteines which form one of the three highly conserved disulfide bonds (Cuff et al. 1998) are spatially separated from each other due to the additional amino acids, it is uncertain if they are still able to build a disulfide bridge within NlH2 and RtH2. The disulfide bridges, however, are necessary for the oxygen-binding capacity of hemocyanins which has been shown to get lost completely after disulfide bond reduction (Topham et al. 1999). If FU-g in this hemocyanin isoform of Muricidae, for example, has lost its oxygen-binding site, this would most probably have reduced the evolutionary constraint on it which furthermore could open the way for the loss of this functional unit within the gene. Such losses of single FUs are facilitated by the modular structure of the hemocyanin genes and occurred several times within the evolution of molluscs (e.g., Cephalopoda (Miller et al. 1990) and Planorbidae (Lieb et al. 2006)). Losses of evolutionary constraint as described in the hypothetical scenario above could be the driving force within the evolution of hemocyanins with varying FU composition. For example, within Cerithioidea, another group of Caenogastropoda, the loss of FU-g and FU-h most probably facilitated the evolution of their mega-hemocyanin. This, however, presumably enabled adaptation to new habitats that could otherwise not be colonized (Lieb et al. 2010; Gatsogiannis et al. 2015) and thus may have had a great impact on the evolution and the large diversity of this group of gastropods.

## Supplementary Information

Below is the link to the electronic supplementary material.

Electronic supplementary material 1 (PDF 173 kb)

Electronic supplementary material 2 (PDF 556 kb)

Electronic supplementary material 3 (PDF 697 kb)

## Data Availability

The cDNA sequences obtained during the current study are available in NCBI under the following accession numbers: NlH1: MT939254; NlH2: MT939255; RtH1: BK014286; RtH2: BK014287.
