# Supplementary material for: Hemocyanins of Muricidae: New ‘Insights’ Unravel an Additional Highly Hydrophilic 800 kDa Mass Within the Molecule
Source: J Mol Evol. 2021 Jan 13;89(1):62–72. doi: 10.1007/s00239-020-09986-6 (PMC7884596; doi:10.1007/s00239-020-09986-6)
Supplement: Supplementary file 1 — Electronic supplementary material 1 (PDF 173 kb) [file 239_2020_9986_MOESM1_ESM.pdf]

Schäfer G. G. (schaefga@uni-mainz.de), Grebe L. J., Depoix F., Lieb B.
Journal of Molecular Evolution

Supplement 1: Sequence comparison and confirmation of RtH1, RtH2, NlH1 and NlH2

Mapping the total transcriptomic raw data to the assembled hemocyanin coding sequences of RtH1, RtH2, NlH1 and NlH2 with low sensitive settings did not reveal any indications for misassemblies.

Furthermore, alignments showed that non-paralogous FUs shared identities below 70%. For paralogous FUs and for the complete coding sequences (CDS) of the two separate isoforms of each species, identities lie below 80% (supplementary table 1). Sequence alignments of RtH1/RtH2 and NlH1/NlH2 revealed that at least every 50 nucleotides hemocyanin cDNA sequences of these two species differ in a nucleotide. Since this value is smaller than the nucleotide overlap used for sequence assemblies (see *methods* section), we can most likely preclude misassemblies.

Supplementary table 1: Identities between the complete coding sequences (CDS) and the paralogous FU-coding sequences (FU-a, FU-b, …) of the two separate hemocyanin isoforms of (i) *Rapana venosa* and (ii) *Nucella lapillus*.

	**CDS**	**FU-a**	**FU-b**	**FU-c**	**FU-d**	**FU-e**	**FU-f**	**FU-g**	**FU-h**
**RtH 1/2**	75%	74%	78%	74%	75%	79%	78%	72%	75%
**NlH 1/2**	69%	70%	77%	73%	73%	76%	76%	66%	75%
