# Supplementary material for: Hemocyanins of Muricidae: New ‘Insights’ Unravel an Additional Highly Hydrophilic 800 kDa Mass Within the Molecule
Source: J Mol Evol. 2021 Jan 13;89(1):62–72. doi: 10.1007/s00239-020-09986-6 (PMC7884596; doi:10.1007/s00239-020-09986-6)
Supplement: Supplementary file 2 — Electronic supplementary material 2 (PDF 556 kb) [file 239_2020_9986_MOESM2_ESM.pdf]

Hemocyanins of Muricidae: New ‘insights’ unravel an additional highly hydrophilic 800 kDa mass within the molecule

Schäfer G. G. (schaefga@uni-mainz.de), Grebe L. J., Depoix F., Lieb B.

Journal of Molecular Evolution

Supplement 2: Amino acid sequence alignment. The alignment includes the full-length amino acid sequences of hemocyanins of the following species: *Megathura crenulata* (KLH, from keyhole limpet), *Lymnaea stagnalis* (LsH), *Melanoides tuberculata* (MtH400), *Nautilus pompilius* (NpH), *Nucella lapillus* (NlH) and *Rapana venosa* (RtH). Shading shows their similarity conservation with *black = 100%* and *grey ≥ 80%*. Positions 2,645 – 2,985 include the additional amino acid sequence sections within NlH2 and RtH2. They lie between two cysteines which typically build a disulfide bridge but are separated from each other within NlH2 and RtH2 due to the additional amino acids (highlighted in yellow within the consensus sequence line). The additional cysteine in FU-d of NlH1+2 and RtH1+2 is highlighted in pink at position 1,348.

```
                 *        20         *        40         *        60         *        80         *       100         *       120         *       140         *       160         *
KLH1    : --------MLSVRLLIVVLALANAENLVRKSVEHLTQEETLDLQAALRELQMDSSSIGFQKIAAAHGAPASCVHK-DTSIACCIHGMPTFPHWHRAYVVHMERALQTKRR-TSGLPYWDWTEPITQLPSLAADPVYIDSQGGKAHTNYWYRGNIDF------LDKKTNRAVDDRL :  159
KLH2    : ----MWTILALLTATLLFEGAFSVDTVVRKNVDSLSSDEVLALEKALDDLQQDDSNQGYQAIAGYHGVPTMCVDKHEKNVACCLHGMPSFPLWHRLYVVQLERALIRKKA-TISIPYWDWTSELTHLPELVSHPLFVGTEGGKAHDNSWYRADITF------LNKKTSRAVDDRL :  164
LsH1    : -----MALLWSFLALALFVLSFGDAALVRKNVDNLSEEDIINLQKTLRDVADDKSAKGYAAIAAYHGYPAQCKDANNRPVACCVHGMPVFPQWHRLYVVQLEQALKEKGL-SIGIPYWDWTRPLTKLPALVSQQVFIESDGTKAKKNVWYQGDIEVVENSKTVIRHTARALDDRL :  169
LsH2    : ---------MLLLLQLAALLSFSGAVLIRKNVELLNPEEVLDLQKSLRSVADDKSNKSYNALAAYHGYPAMCEHQ-GKPVACCVHGMPVFPQWHRLFVVQFEQALKDKGL-SIGVPFWDWTKPLADLPELVRQQVFIDSNGGKAKKNVWYQGSIPL----KTGPRFTARAVDDRL :  160
MtH400  : --MVTSPRFALALLAFCVQVAFTTGSLIRKNVENLSSQEILSLQTSLRALAADESASGFKAVAAYHGEPSMCEDNDGVAIACCLHGMAVFPQWHRLYVVQFEQLLVNKGL-NIGVPYWDTLEPLTHLPSLVEEQIFRDPNGGVGGKNSWFSGEVPV----DGAIKNTARAVDDRL :  168
NlH1    : ---MTSPQVTGTLLFSLAFLCLTTGSLLRKNVDSLTEQEILRFQSTLMELENDHTDHGFQALGGYHGEPSGCHMKDGAEIACCIHGEATFPMWHRAYTVQFEQLLVMKGLSNLGVPYWDWSEPLTSLPELVRHQIFKDPEGGVGQRNAWFSAEINH----GDVHEHTARAVDDRL :  168
RtH1    : ---MTSPRVTGTLLFCLAYLCFATGSLLRKNVDTLTEQEILRFQNTLLELEKDNSDHGFQALGAFHGEPSGCQTKEGSAIACCVHGEATFPMWHRAYTAQFEQLLVEKGLANLGVPYWDWTRELTHLPELVRHQIFKDPNGGVGQRNAWFSAEIHH----GDVDEHTARAVDERL :  168
NlH2    : ----MTPRVLATLVWGVAYLCLSTGSLVRKSLDSLTPQEILTLQTTLRALENDETDHGFQALAAYHGEPSQCQTADGQLIACCHHGDPTFPLWHRAYVVQFEQALVEKGLSNLGVPYWDWTEPLTHLPHLVEHQIFRDPAGGLGQKNAWFTADINH----GEVHEHSARAVDERL :  167
RtH2    : ----MTPWVFQALVWCVAYLSLATGSLLRKNVDSLTEEEILTLQSVLRELENDQTEHGFQTLAAFHGEPSQCALRDGRLIACCRHGDPTFPLWHRAYTVQFEQALVQKGLANLGVPYWDWTEPLKDLPELVRHQIFRDPKGGLGQKNAWFTADIEH----GDVHEHTARAVDDRL :  167
NpH     : MATHWHSLLLFSLQLLVFTYATSDPTNIRKNVADLTHDVALNLQIALRTMQDDNSPIGFQAVAAYHGEPASCIDSHENLVVCCLHGMPTFPHWHRLYTTHLEQSLLALGT-TLGLPYWDWTQPMTHLPNIVQHPLFIDPNGGKAKKNVFYSGTIGF------KKMQTARAVDERL :  168
                        l                  6RKn6 L   e 6 l2  L  6  D 3  g5 a6aa HG P  C       6aCC HG   FP WHR 5 vq Eq L  kg     g6P5WDw  p6t LP 6v   65 d  Gg     N 55    6             3aRA6D RL

           180         *       200         *       220         *       240         *       260         *       280         *       300         *       320         *       340         *
KLH1    : FEKVKPGQHTHLMESVLDALEQDEFCKFEIQFELAHNAIHYLVGGKHDYSMANLEYTAYDPIFFLHHSNVDRIFAIWQRLQELRNKDPKAM------DCAQELLHQKMEPFSWEDNDIPLTND----------------YDTLNLNGMTPEELKTYLDERSSRARAFASFRLKGF :  312
KLH2    : FEKVQPGHHTRLMEGILDALEQDEFCKFEIQFELAHNAIHYLVGGRHTYSMSHLEYTSYDPLFFLHHSNTDRIFAIWQRLQQLRGKDPNSA------DCAHNLIHTPMEPFDRDTNPLDLTREHAKPADSFDYGRLGYQYDDLSLNGMSPEELNVYLGERAAKERTFASFILSGF :  333
LsH1    : FQKVEPGQNTDLFEQVLNALMYPNYCQFEVQFEIAHNTIHYLVGGRNKYSMSHLEYTSYDPIFFLHHSNVDKIYSIYEALQKEKGYTPGPGCTGKCELCDLKHFDEPLAPFSWETNPFAVTKQFNLPREAVDGSIYGYKYDNLTLNGYDLNGIKRLLKEKWSHAASYAVFRLSGI :  344
LsH2    : FQKVEAGEHTDLFEQVLNALEYPNYCQFEVQFEVAHNTIHYLVGGRNRYSMSHLEYTSYDPIFFLHHSNVDRIYAVYEDLQRLRGYTPGPGCKGECETCDIKGFQKPLEPFNRASNPWPRTRNHATGFESLDHVLFGYQYEDRTLNGHDVDEIFRILKERQKHDRAFAAFKLAGI :  335
MtH400  : FEKVAPGKNTQLFNIILDALEQDDYCQFEVQLEVAHNHIHYLVGGRHQYSMATLEYSSYDPIFFLHHSNIDRIFVIWQELQKKRGKPYDHS------DCSVEQFRWNLKPFDRDTNPIALTKQFHTAKELFSNLQLGYVYDDLTLNGMDVDQLYNLMEQRRARERAFGVFSLHGL :  337
NlH1    : FEHPEEGHNTRLFDLILDALEQHDYCHFEAQLEVGHNHIHYLVGGRHHYSMATLEYSAYDPIFFLHHSNVDRIFVIWQEMQKRRGLPYDHS------DCSVELFNKPMEPFKRDSNPVQITKTYSKPRDLFSHLQLGYTYDDLSLGGMSLDELYHHLEERSGRDRTFASFALHGV :  337
RtH1    : FEHPQDGGKTRLFNMILDALEQDDYCQFEAQFEVAHNHIHYLVGGRHHYSMSTLEYSSYDPIFFLHHSNVDRLFVIWQEMQKRRGKPYDHA------DCSVELFNKPMEPFKRDSNPVELTRTFSKPRDLFSHLQLGYTYDDLELGGMSLEELHAYLQQRHGRDRTFATFALHGL :  337
NlH2    : FEHVEPGQKSRLFEMTLDALEQDGFCHFEAQFEVAHNHIHYLVGGRHHYSMSTLEYTAYDPIFFLHHSNVDRLFIIWQELQKLRGKPHDHS------DCGVELFNKEMEPFKNDNNPVELTRTFSKAKNLFSHLQLGYTYDTLELNGMSIKQLHSLLEHRRGRDRHFAAFSLHGV :  336
RtH2    : FEHVAPGGKTRLFEMILDALEQDEFCHFEAQFEVAHNHIHYLVGGRHHYSMSTLEYTSYDPLFFLHHSNVDRLFVIWQELQKKRAKPYDHS------DCGVELFGKPMEPFNHDNNPVELTKTFSKPKELFSHLQLGYTYDTLSLNGMTIDQLHTLLEYRRGRDRKYAAFSLHGL :  336
NpH     : FSQPEPGHHTFLFEGILDALEQTDYCQFEVQFEIIHNAIHYLVGGQFPHSMSSLEFTSYDPLFFLHHSNVDRLFSIWQALQRHRGLPSGHS------NCAKELFTVPMKPFNEDSNPIQLTHDHALPSQLFDHTKLLYQYDDLTLNGLNITQVQELIEKRRSHARAFASFRLSGI :  337
          F     G  3 Lf   L1ALeq  5C FE QfE6aHN IHYLVGG   ySM  LE53 YDP6FFLHHSN D465 652 6Q  4g          C     f   6 PF    Np   T                 gy Yd l LnG      6    6  4     r 5a F L G

                 360         *       380         *       400         *       420         *       440         *       460         *       480         *       500         *       520
KLH1    : GGSANVFVYVC----------IPDDNDRNDDHCEKAGDFFVLGGPSEMKWQFYRPYLFDLSDTVHKMGMKLDGHYTVKAELFSVNGTALPDDLLPH----PVVVHHPEKGFTDPPVKHHQ-------SANLLVRKNINDLTREEVLNLREAFHKFQEDRSVDGYQATAEYHGLPA :  466
KLH2    : GGSANVVVYVC----------RPAHDEISDDQCIKAGDFFLLGGPTEMKWGFYRAYHFDVTDSVASIDDDGHGHYYVKSELFSVNGSALSNDILRQ----PTLVHRPAKGHFDKPPVPVA-------QANLAVRKNINDLTAEETYSLRKAMERFQNDKSVDGYQATVEFHALPA :  487
LsH1    : STSANVRVQVC---------VPSEDEVTGTYCEFAGDFFVLGGPLEMPWAFSRPYYFEVTKTVEKLGLALDGNYRIEAELFSVNGTQLPSSLLPH----PFVSYRPAKGTVDTPIAHNEVNHEHEYHEGISVRKDIDRLTNEEIYELREALQKFQNDKSVDGYQAIAEFHGDPG :  505
LsH2    : GTSADVKVKVC----------LPALDAVTEDHCEDAGHFFILGGPLEMPWRFSKPYYFDITKTVASLGLPLDGNYHVEAEIYSVNGTLLPPGLLPA----PYVSYRPAKGKIDPVIEISGPDHD---HEKVAVRKNVDRLTDVEIYELRQALKKFQNDKSVDGYQAIAEFHGDPG :  493
MtH400  : GFSANARVKVCSDLTGRRKRTIGSADLDVEHHCEFAGDFFLLGGANEMPWEFHLPFYFDVSDAVQRLGLSLTSKWSLEWDLYHVNGTALPGNLLPT----PYGKHRPAKGDRDSEEVSFFADRDV--HRHAVVRKNVVDLTDEEVYELRLAMDRFQNDTSVDGYQAIAEFHGLPA :  506
NlH1    : GFSANVRVKVC-DVHDDGDAKDYYGNDDHHDHCVLSGDFFILGGANEMPWEFTVPVLFDVTDAIHNLTLEEGGHYGVHVEVYSVNGTRLGAHVLGTT---PHLSHRPARGYHDPEIPDGR-------SGKTVVRKDVEVLTKEEMYHLRQAMAKFQNDTSIDGFQAVAEFHGLPA :  501
RtH1    : GFSANVRVKVC-DVHNDGERQGDAASQAEEDHCEFSGDFFILGGANEMPWEFTVPYYFDVTEAVHKLKLGPGGHYGVKADVYSVNGTLLGAHVLGSR---PYLSHRPAEGYHDPEIHPDL-------KKTTLVRKDVETLTDEEVYYLRQAMAKFQNDTSIDGFQAVAEFHGLPP :  501
NlH2    : GFSANVRVKIC------------YMEDEEEDHCEFSGDFFILGGKNEMPWEFTVPYYFDVSETMDHLRHGRSKLHG-HFEVKAEVYSVNGTRLESDILSKPYISHRPAHGFTDPVIHHSR-------RATTVGRKNVDVLTDEEVYHLRQALARFQNDTSIDGFQAVAEFHGLPA :  491
RtH2    : GFSANVRVKIC------------HVETEEEDHCEFSGDFFILGGSNEMPWEFTVPYYFDVTETMEHMTYGLDEDHGGHLEVKAEVYSVNGTLLGSQILGKPYISHRPAHGFTDPVIHRNR-------GTTTVGRKDVEVLTDEEVYHLRQALARFQNDTSIDGYQAVAEFHGLPA :  492
NpH     : HTSANVRVHVC---------VRIDDDKSHDDCDHVAGNFFILGGAREMEWHYHYPYRFDITSTVQELGIALDSNYYVDIDLIAINGTLLSSSLIPN----PTVDYQPAPGSSDPPMRQID-------REKLVVRKDLRRLHDTEEFGLRQAMARFMKDESIDGFQALAEYHGLPA :  492
          g SA1v V 6C                    d c   G FF6LGG  EM W 5  p  Fd63  6  6              6   ng3 l   6     P   rPa G  D                      vRK16   Lt eE   LR A  4FqnD S6DG5QA aE5HglP

              *       540         *       560         *       580         *       600         *       620         *       640         *       660         *       680         *       700
KLH1    : RCPRPDAKDRYACCVHGMPIFPHWHRLFVTQVEDALVGRGATIGIPYWGLPYWDWTEPMTHIPGLAGNKTYVDSHGA--SHTNPFHSSVIAFEENAPHTKRQIDQR-LFKPATFGHHTDLFNQILYAFEQEDYCDFEVQFEITHNTIHAWTGGSEHFSMSSLHYTAFDPLFYFHH :  638
KLH2    : RCPRPDAKDRFACCVHGMATFPHWHRLFVTQVEDALLRRGSTI-----GLPNWDWTMPMDHLPELATSETYLDPVTGE-TKNNPFHHAQVAFENGVTS--RNPDAK-LFMKPTYGDHTYLFDSMIYAFEQEDFCDFEVQYELTHNAIHAWVGGSEKYSMSSLHYTAFDPIFYLHH :  653
LsH1    : KCPNPNSKNRKACCIHGMPTFPHWHRLLVVQVEDALRRRGSNI-----GVPYWDWTKPRTSVPKLAADETYTDPTTNE-VKRNPFHDAPIAFLGDSTKTTRDVSSS-LSDSPKWGDHTELFDAFLLALEQDNFCDFEVQFEVAHNLIHALVGGKEQYSLATLSYSAFDPIFYLHH :  673
LsH2    : KCPNPTAKDRMACCVHGMPTFPHWHRLLVVQVEDALRRRGSPI-----GIPYWDWTRPNTKIPDLANDVKYIDPKTKD-ELDNPFHDAEISFLDNNVHTSRDVQKD-LSQTPLWGDHTELFDAFLLALEQDNFCDFEVQFEIAHNLIHGLVGGNSKYGLSSLSYSAFDPIFYIHH :  661
MtH400  : RCPRPDAAVRYACCIHGMATFPHWHRLFVVQIEDELKARDLHF-----GMPYWDWTVPNAQIPAIAAEATYQDPHHPDVTLHNPFYDAAVAFLNTRTT--RDVQND-LAENPIYGDHSKLYDGILLAFEQTDFCDFEIQFEVIHNSPHFLVGGFGAYTLATLHYSAFDPIFYLHH :  673
NlH1    : KCPHPDAAVRYACCVHGMPTFPHWHRLFVTEVEDELKSRGLEI-----GMPYWEWTRPNTHVPELAAEETYEDPHTHH-QVHNPFHDAVVAFLGEKTS--RDIQEDELSETPDFGDHTSLFDAMLLAFEQEDFCDFEVQFEVVHNAIHFLVGGLNPYTMATLHYSAYDPIFYLHH :  668
RtH1    : KCPHPDAAVRYACCIHGMATFPHWHRLFVTEVEDELKSRGLEF-----GIPYWDWTRPNTHVPALAAEETYEDPHTHH-QVHNPFHDALVAFLKKKTT--RDVQAD-LTETPAFGDHTALFDGMLLAFEQTDFCDFEVQFEVVHNAIHFLVGGFDPYTMATLHYSAYDPIFYLHH :  667
NlH2    : KCPHPDAAVRYACCLHGMPTFPHWHRLFVTEVEDELRSRGLDI-----GIPYWDWTEPETHVPALANDETYEDPHTHE-QLHNPFHDAPIVFLGEKTT--RQIQDE-LSEKPKFGDHTALFDGMLLAMEQTDYCDFEVQFEVVHNAIHFLVGGFGPYTLTTLHYSAFDPIFYLHH :  657
RtH2    : KCPHPDAAVRYACCIHGMPTFPHWHRLFVTEVEDELRSRGLEI-----GIPYWDWTKPNAHVPALANDETYEHPYTHE-TIHNPFHDAAVAFLGQRTT--RQIQDD-LAETPKFGDHTTLFDGMLLAFEQTDFCDFEVQFEVVHNAIHFLVGGFGPYTMATLHYSAFDPIFYLHH :  658
NpH     : RCLTPEG-NRIACCIHGMATFPHWHRLFVLQVEDALIRRGSPI-----GIPYWDWARPMISLPHLIADETYINPYSNK-SVPNPFHHGSIDFLHDHITTQRDIKQETLFEQPAFGDHTYLFDGLMYALEQDDFCDFEIQFEIVHNGIHAWTGGQQTYSMSSLHFTSFDPLFFLHH :  660
          4Cp P a   R ACC6HGM tFPHWHRLfV 26ED L  Rg  i      G6PyWdWt P   6P 6a   tY  p         NPFh a 6 Fl       R     L   p 5GdH3 L51  6 A EQ 15CDFE6Q5E6 HN iH  vGG     5 6 3Lh53a5DP6F5 HH
```

```
                 *       720         *       740         *       760         *       780         *       800         *       820         *       840         *       860         *
KLH1    : SNVDRLWAVWQALQMRRHKPYRAHCAISLEHMHLKPFAFSSPLNNNEKTHANAMPNKIYDYENVLHYTYEDLTFGGISLENIEKMIH-ENQQEDRIYAGFLLAGIRTSANVDIFIKTTDSVQ-HKAGTFAVLGGSKEMKWGFDRVFKFDITHVLKDLDLTADGDFEVTVDITEVD :  811
KLH2    : SNVDRLWAIWQALQIRRGKSYKAHCASSQEREPLKPFAFSSPLNNNEKTYHNSVPTNVYDYVGVLHYRYDDLQFGGMTMSELEEYIH-KQTQHDRTFAGFFLSYIGTSASVDIFINREGHDK-YKVGSFVVLGGSKEMKWGFDRMYKYEITEALKTLNVAVDDGFSITVEITDVD :  826
LsH1    : SNVDRIWAIWTALQELRGKPYKAHCAQSYTYEPLKPFAFSSPYNNDQKTFTHAVPRNVYDYETELNYAYDSLEFGGLTIKQLNDYINNERKTKNRVFAGVLLWGIKRSALATLYVND------VEAGRFALLGGPSEMEWRFDRLYRHEITDTLEELNLHWAQPYTVTLKLTDFE :  842
LsH2    : SNVDRIWAIWTALQQHRGKPYKAHCAQSYTYEPLKPFAFSSPYNNNEKTFSHSTPRNVYDYEAELGYAYDSLEFGGMAIPELDDFINSNLKGRSRVFVGILLSGIKKSGIAKIKVITMRNEE-YEAGRFALLGGPSEMPWSLDRVYRHDITTALEKMGLFWAQPYEVKLEMTEFD :  835
MtH400  : SNVDRMWAIWQELQIRRGLPYRAHCAHTMTHEPMKPFSFAAPLNNNEKTFSHSEPSQVYDYERDLGYTYDSLTFGGMTIEQLENYLE-ERQSKERVFVGIELHNIGASAWVKVLIDSGSGGEPYLVGKIAVLGGEKEMPWHFDRLFKMEITDALSDLGLSYDDNFDVTLQITDVT :  847
NlH1    : SNVDRLWAIWQRLQMRRGKLYKAHCAGSLTEEVMKPFGFPPPYHNDPKTHDNARPDKVYDYEHVLEYTYDSLLFGGMTIEQLDHYLE-ERQTHDRVFVGVMLHNIGISAWATLSIEMKNGEN-YTVGKLGVLGGEKEMPWHFDRPYKVEITDALHKLGYRYDDDFHVHMHLTDVK :  841
RtH1    : SNVDRLWAIWQKLQMRRGKLYKAHCAGSLTQELMMPFGFPPPYHNDPKTHDNARPSQIYDYESVLDYTYDSLQFGGMTIAQLDHYLE-ERKTHDRTFVGIMLHNIGISAWATLAIEMKNGEE-YTVGKVAVLGGEKEMPWHFDRTFKVEITEALHKLGYRYDDEYDVKLHIKDVT :  840
NlH2    : SNVDRLWAIWQELQMRRGLPYKAHCAGSLTYEVMKPFGFLPPYHNDAKIHDNARPYKIYDYEKTLDYAYDSLQFGGMTIEQLDHYLE-ERKSHDRAFVGIMLHNIGISAWATLSIEMTNGEK-YEVAKLAVLGGEKEMPWRFDRAYRVEITDALRHLGFSYDDEFHVHLDIIDVT :  830
RtH2    : SNVDRLWAIWQELQMKRGLPYKAHCAESLSKEVMKPFGFPPPYHNDQKIHDNARPFKIYDYESVLDYTYDSLQFGGMTVDQLDPYLE-ERKTHDRVFVGIMLHNIGVSAWCTLSIMRASGDK-YEVTKLAVLGGEKEMPWRFDRAFKVDITEALKTLGFSYDDDFHVHLDITDVT :  831
NpH     : SNVDRIWAIWQALQIYRGKHYLAHCANSEVHSPMRPFSFPSPRNPNPRTFTHATPTDIYDYEAELGYTFDTLDFGGRSIPDLQNEIE-EHQRHDRTFAGFLFWGIHTSTNGKVFVDVIGQAT-YNAGTIAILGGTKEMTWRFDRLYHFEISEALKALGKTADDEFSLHVKIHDVN :  833
          SNVDR6WA6Wq LQ  Rg  Y AHCA 3   e 6 PF F  P  n1 4t     P  6YDYe  L Y 5d L FGG  6   6    6  e     R 5 G  l  I  Sa   6 6             y  g  a6LGG kEM W fDR 5    I3 aL  6     d  5 6 6 6 dv

            880         *       900         *       920         *       940         *       960         *       980         *      1000         *      1020         *      1040         *
KLH1    : GTKLASSLIPHASVIREHARGKLNRVKFDKVPRSRLIRKNVDRLSPEEMNELRKALALLKEDKSAGGFQQLGAFHGEPKWCPSPEASKKFACCVHGMSVFPHWHRLLTVQSENALRRHGYDGALPYWDWTSPLNHLPELADHEKYVDPEDGVEKHNPWFDGHIDTVDKTTTRSVQ :  986
KLH2    : GSPPSADLIPPPAIIFERADAK-------DFGHSRKIRKAVDSLTVEEQTSLRRAMADLQDDKTSGGFQQIAAFHGEPKWCPSPEAEKKFACCVHGMAVFPHWHRLLTVQGENALRKHGFTGGLPYWDWTRSMSALPHFVADPTYNDAISSQEEDNPWHHGHIDSVGHDTTRDVR :  994
LsH1    : GHDIPINPFPKPQIIFKAKEEP-----KDVSADTIKIRKNVATLTEAEVVDLRQALANLQNDQSAGGYQDLGRFHGTPNWCPTPDAEVKKSCCLHGMPTFPHWHRLWTVQAENALRRHGYVGAIPYWDWTQFVKSLPDLVTSEKYRDPSSNNEIKNPFYSAHITDADQDTVRSPR : 1012
LsH2    : GTPIDVRLFPRIQLIYRAPEHP-----KAAAEHDVHVRKNVDQLTEEEVHDLRQALSNLQDDKSNGGYQDLGRFHGTPKWCPSPEAEKKVSCCLHGMPTFPHWHRLHAVQAENALRRHGYHGGLPYWDWSRPLKSLPALVSSPTYLDPSNNEQVKNPFYSAHIDDVDHDTVRSVR : 1005
MtH400  : GKEWSSDSFQHDTIFHEPGKTF-----EVRGVSDKRTRLDVDHLNSEQIQNLRDAFKALEDDTSINGFNQIAAFHGQPSWCPSPDAETKYACCLHGMPTFPHWHRLLITVQLENALRDKGFESGLPYWDWTLPMTELPESFTAETYDNPVSGANEANPLHHAVVE--GHKTTRSVR : 1015
NlH1    : GHEWPEDTFSHHTIFHQPASIH----HEEEYHKDEHVRMDVETLTVEQEQYLREALATLKHDHSISGYNQIASFHGQPNWCPSLEAEHKFACCTHGMPIFPHWHRLLTVQAENALRAHGLHSGLPYWDWTLPMTELPKFVAVATYESPRTHHEEINPWYNAEVN--DHNTTRSVR : 1010
RtH1    : GKEWPEDTFSHHTIIHVPASTH----LEEDHKEEDHVRNDVDTLTKEQIQNMREALATLKQDHSPGGFDHLAAFHGQPNWCPSMQAEHKVACCPHGMPVFPHWHRLLTVQAENALIAHGMHSGLPYWDWTLPMTALPKFVADATYENPKTHHTEPNPWFSGEVD--GHNTSRMVR : 1009
NlH2    : GKKWPEDTFSHETIIHVPAEVH----TEDNFEKDDHVRNDVEHLTVEQMQNLREALHSLKEDHTISGYNQIAAFHGQPNWCPSPQAEKKFSCCAHGMAIFPHWHRLMTVQMENALMANGMITGLPYWDWTLPMTELPFLVREATYHNPKTDLDEPNPWYSGVVD--GHETHRNVR :  999
RtH2    : GKKWPKDTFSHDTIIHVPASAK----AEDDFASDDHVRSDVEHLTAEQMQNLREALRALMNDASIAGYNQIASFHGQPNWCPSPDAEEKFACCAHGMPIFPHWHRLLTVQAENALMAHGMTSGLPYWDWTLPIDHLPELVAGENFDNPKNGQSEPNPWFSGEVN--GHETQRNVR : 1000
NpH     : GSRLSNDIIPAPNIIFVPAHVK-----SMNISHKGHTRRNINSLTQESLYELRQALTSFMADTENTGFQRYAAFHGFPEWCPSPDAEHKFACCVHGMPTFVHWHRLLTIQFENGIRNHGYQCGLPYWDWTEAITHLPESLTAAKYTDPHSNEMHHNPLHNGHIDSVNADTMRNIN : 1003
          G           6i                            R  6  L  e    6R A   l  D    G5       FHG P WCP3p Ae K   CC HGM  FpHWHRL t6Q  ENa6  hG   g6PYWDW3 p6  LP           5  p         NP     6        T R  r

              1060         *      1080         *      1100         *      1120         *      1140         *      1160         *      1180         *      1200         *      1220
KLH1    : NKLFEQPEFGHYTSIAKQVLLALEQDNFCDFEIQYEIAHNYIHALVGGAQPYGMASLRYTAFDPLFYLHHSNTDRIWAIWQALQKYRGKPYNVANCAVTSMREPLQPFGLSANINTDHVTKEHSVPFNVFDYKTNFNYEYDTLEFNGLSISQLNKKLEAIKSQDRFFAGFLLSGF : 1161
KLH2    : DDLYQSPGFGHYTDIAKQVLLAFEQDDFCDFEVQFEIAHNFIHALVGGNEPYSMSSLRYTTYDPIFFLHRSNTDRLWAIWQALQKYRGKPYNTANCAIASMRKPLQPFGLDSVINPDDETREHSVPFRVFDYKNNFDYEYESLAFNGLSIAQLDRELQRRKSHDRVFAGFLLHEI : 1169
LsH1    : DDLFEQPKFGQNTAIAKQVLLALEQDNFCDFEVQFEIAHNFIHALVGGNEQFSMASLLYTAFDPLFYLHHANTDRLWAIWQALQKYRNKPYNTANCAIGQLRKPLSPFSLTSSVNPDANTREHSLPFQVFDYRNNFHYEYDNLEFNGLSIPQLAKELEKNKGDDRVFVGFMLHGI : 1187
LsH2    : SDLFQQPGYGEYTDIAEQILLALEQDNFCDFEVQFEIAHNFIHALVGGDKEPYSMASLMYTAFDPLFYLHHSNTDRIWAIWQALQNYRGKPYNTANCAIGQLRQPLAPFSLTSDVNPDAVTREHSVPFQVFDYRNNFHYTYDNLEFNGLTIAQLARKLEQNKDSDRVFAGFMLHGI : 1180
MtH400  : GELFQQPAFGKFTRIGEQVMLAFEQTDYCSFEVQFEVAHNYIHALVGGNELYSMASLRYTAYDPLFALHHSNTDRIWAIWQALQKYRGLPYNTANCAITQLRKPLQPFAQTSTVNPDSVTRDHSVPFDVFDYSSSFHYDYDNLEFNGLSIAQLQHEVVVRKAHERVFAGFMLHGI : 1190
NlH1    : DELFQQPELGKLTRIAEKVMLAFEQDNFCDFEIQYEIAHNHIHALVGGNQLYSMASLRYTAFDPIFYLHHSNTDRIWAIWQVLQKIRGKPYNSANCALSELRKPLQPFAQTSVTNPDPVTRDNSAPFKVFNYRKSFHYRYDNLQFNGMSPPQLQREIIKRKGLERVFAGFMLHGI : 1185
RtH1    : DDLFEQPEFDKMTRIAEKVMLAFEQDNFCDFEIQYEIAHNHIHALVGGNKLYSMASLRYTAFDPLFFLHHSNTDRIWAIWQTLQKMRGKPYNSANCAIAELRKPLQPFAQTSVTNPDPVTRDHSVPFDVFKYHENFHYRFDNMQFNGMSPPQLLRETVRRKGLDRVFAGFMLHGI : 1184
NlH2    : EDLFKQPDFGHMTPLAEKVMLAFEQDNFCDFEIQYEIAHNYIHAVVGGTEEYSMASLRYTAYDPLFFLHHSSTDRIWAIWQHLQKFRNKPYNSANCAIAELRRPLQPFAQTSLTNPDPVTRDHSVPFDVFSYHDSFHYEYDNLQFNGMSIPQLHRETVKRKGLSRVFAGFMLHGI : 1174
RtH2    : DDLFKQPAFGEMTPLAEKVMLAFEQDNFCDFEIQFEIAHNFIHAVVGGTELYSMASLRYTAFDPLFYLHHSSTDRLWAVWQKLQKFRNKPYNSANCALAEMREPLQPFAQTSATNPDPVTRDHSVPFDVFNYRNSFHYEYDNFQFNGMSIPQLQRETLKRQGLDRVFAGFMLHGI : 1175
NpH     : EKLFEQPEFGHFTLLANQVILALEQENFCDFSVQFEISHNEIHALVGGTEPYSMASLTYTAFDPLFMLHHSNVDRIWAIWQSLQQYRGHPHKSANCALQQLHQPLSPFSLTSEVNPDPETREHSKPMDVFDYEHNFHYHYDNLELLGMSIPQLSREINRRRSRNRVFAGFMLHGF : 1178
           L5 qP  g  T 6a   66LA  EQd15CdFe6Q5E6aHN  IHA6VGg    5sMaSL YTa5DP6F  LHhsntDR6WA6WQ  LQk R  kPyn  ANCA6   6r  PL PF    s  NpD   T4 hS  Pf  VF  Y    FhY  5dn   fnG63i  QL   e          RvFaGF6Lhgi

             *      1240         *      1260         *      1280         *      1300         *      1320         *      1340         *      1360         *      1380         *      1400
KLH1    : KKSSLVKFNICTD-SSNC-HPAGEFYLLGDENEMPWAYDRVFKYDITEKLHDLKLHAEDHFYIDYEVFDLKPASLGKDLFKQPSVIHEPRIGHHEG--EVYQAEVTSANRIRKNIENLSLGELESLRAAFLEIENDGTYESIAKFHGSPGLCQLN-GNPISCCVHGMPTFPHWHR : 1331
KLH2    : GQSALVKFYVCKHNVSDCDHYAGEFYILGDEAEMPWRYDRVYKYEITQQLHDLDLHVGDNFFLKYEAFDLNGGSLGGSIFSQPSVIFEPAAGSHQA--DEYREAVTSASHIRKNIRDLSEGEIESIRSAFLQIQKEGIYENIAKFHGKPGLCEHD-GHPVACCVHGMPTFPHWHR : 1341
LsH1    : KRSALIKFQIHKN-DTEF-KEAGEFYILGDENEMEWSYDRLYKYEVTQQLEDFGLRYNDRYYIKYQVLDLDGSIYSTDTFSTPTVLFELGTSNLYG--KEFRAVSTAASQIRRNLDSLSQGEIESLRAAFLSIKEDGTYEKIASFHGKPGKCKDSAGRTVACCVHGMPTFPAWHR : 1358
LsH2    : GKSALVKFYICRS-ADDCNNYAGEFYILGDPHEMEWAYDRLYKYEITRQLSDLNLHYNDRYYIRYEVLELSGEVFSTNVFPTPTILYDLGTSHLYG--KEYRKPVTAASQIRRDLETLSKGEIESLRSAFLSIKADKTYEKIASFHGKPGLCEHN-GHKVACCVHGMPTFPAWHR : 1351
MtH400  : KQSALVRFEICSS-GGAC-GQAGEFYILGDDYEMPWEYDRLFKYEITDQLKEQGLEPLDRYDIKYTVFALDGSVVGTDTFGQVTVVHTYGAGHVER--KSYGEELKASSHVRRNVDDLTGGEIESLKAALQKMEDDGSFENIAEFHGAPGLCELN-GKKVGCCVHGMATFPHWHR : 1360
NlH1    : QKSSLVVFEICKP-DDTC-KQAGEFYLLGDEFEMPWEYDRLFKYEITDQLKDFDLKPTDRYDIHYTVYDLNRQNLGEDLFGNATIVYKHGLGHMRGHEGDYLEEVMASSHVRKDMSTLTNGECESLRSALHDMEEDGSFEQIAKFHGYPGLCDHK-GRAVGCCMHGSPIFPHWHR : 1357
RtH1    : QKSALVVFEICKP-DGTC-KEAGEFYLLGDEFEMPWEYDRLFKYDITEQLEKFDLEPMDRYDIQYTVSDLNGQNLGDDLFGNATIVYTPGLGHMKGHEEDYRAEVRASSHVRRNLKSLTTGECESLRSALHDMEEDGSFEAIAQFHGSPGLCDHK-GKQVGCCVHGAPTFPHWHR : 1356
NlH2    : KKSSLVVFQICTP-DHTC-TDGGEFYLLGDEYEMPWEYDRLYKHEITSQLEKRGLDHHDRFDVEYHVYDLEENDLGSDVFGNASIVYKPGLGQMKGHAEDYLQEVRASSHIRQNIDSLTLGECESLRSALHDMEEDGSFEKIAKFHGYPGECQHD-GSPSSCCVHGSPTFPHWHR : 1346
RtH2    : KKSSLVVFNICTS-DKSC-TKAGEFYLLGDEFELPWEYDRLYKHEITKELKEMSLDHHDPFDIHYNVYDLENNDLGDDVFGNASIIFKSGRGHLKGHTEDYLREVRASSHIRRNIEALTKGECESLRSALHDMEEDGSFEKIAQFHGSPGLCTHD-NHPAACCVHGSPTFPHWHR : 1347
NpH     : KKSALVSFSILHH-QTDE-TYAGEFYILGSEDEMPWHYDRLYRYEITDILNELHLQYTDPLRITYRINDLHNKVVEGEEFSPTVLYVPALGTYGTQ--TKWREPVTSASRIRKDLNTLTDGELESLRNAFLRLQEEGAYEPIAAFHGVPAKCRGEDGNIYTCCVHGMPTFPHWHR : 1349
            S L6 F 6c       c   aGEFY6LGde  E6pW  YDR654ye6T   L     L   D     6 Y   dL             F    6                       5         s 6R  16   L3 GE  ES64  A     6  dg  5E  IA  FHG  Pg  C      g       CC6HG  ptFPhWHR

                 *      1420         *      1440         *      1460         *      1480         *      1500         *      1520         *      1540         *      1560         *
KLH1    : LYVVVVENALLKKGSSVAVPYWDWTKRIEHLPHLISDATYYNSRQHHYETNPFHHGKI--THENEITTRDPKDSLFHSDYFYEQVLYALEQDNFCDFEIQLEILHNALHSLLGGKGKYSMSNLDYAAFDPVFFLHHATTDRIWAIWQDLQRFRKRPYREANCAIQLMHTPLQPFD : 1504
KLH2    : LYVLQVENALLERGSAVAVPYWDWTEKADSLPSLINDATYFNSRSQTFDPNPFFRGHI--AFENAVTSRDPQPELWDNKDFYENVMLALEQDNFCDFEIQLELIHNALHSRLGGRAKYSLSSLDYTAFDPVFFLHHANVDRIWAIWQDLQRYRKKPYNEADCAVNEMRKPLQPFN : 1514
LsH1    : LYVEQVEEALLGRGSSVAVPYWDWTTAFTKLPDLINQATYFNSRQQRFEPNPFFSGSI--AGEGAVTTRDPQPELFNNDDLYEQVLYALEQDHFCDFEIQFEIIHNALHSLLGGRAQYSLSSLDYSAFDPVFFLHHANTDRLWAIWQELQRYRGLKYNEADCAINQMRTPLRPFN : 1531
LsH2    : LYVEQVEEALLSRGSSVAVPYWDWTKPITKLPELINEATYYNSREQRFDPNPFFCGTI--ANENAVTTRDPQPELFNSEELYEQVLLALEQEHFCDFEIQFELVHNALHTLLGGRSQYSLSSLDYSAFDPVFFLHHANTDRIWAIWQELQRYRGLPYNEADCAINLMRKPLQPFN : 1524
MtH400  : LYVEQVENALLSHGSAVSIPYWDWTKPITKLPDIIAQETYFDSRSQTMQNNPFFRGPIRTPDVNDYTTRDPQPELFNNDYFLQQTLLALEQTSYCDFEAQFEVTHNAFHSFLGGRGKYSLSTLDYSAFDPVFFLYHANTDRIFAIWQALQSYRGLHWDEADCALNHMRDGLHPFD : 1535
NlH1    : LYVEQVENALLNHGSAVSVPYWDWTEHFDELPNLIGKPTYFNSRMHRKEINPFFRWKV--SGSGEFTSRDPRFDLFDSEYFLESAMLALEQTSFCDFEVQFEVTHNAIHSFLGGRGKVSMSTLDYSAFDPIFMLHHSTVDRIWAIWQALQKHRDLSFDETDCAVNLMGQTMHPFD : 1530
RtH1    : LFVEQVENALLDHGSAVSVPYWDWTEDFDHLPKLLAMPTYYNSRMHSKDVNPFFRWKV--SGTDEYTTRDVRFDLFNAEYFLDNILLALEQTSYCDFEVQFEVSHNAIHSFLGGRGKVSMSTLDYSAFDPIFFLHHANMDRIWAIWQALQKHRDLPFDETDCAVNLMSKPLHPFD : 1529
NlH2    : LYVENVENALLNHGSAVSMPYWDWTQPIVHLPKLFSLPTYYDSRTQHKEPNAFFRWKI--PDMDEYTSRDPQPELYDSHYFLDNVLLALEQTSFCDFEVQFEVVHNALHAFLGGRGKYSLSTLDFAAFDPVFFLHHANVDRIWAIWQALQKFRGGSFDETDCAMNLMATPLHPFD : 1519
RtH2    : LYVENVENALLNHGSAVSMPYWDWTQPIEHLPALINEPTYYDSRTHKKEANAFFRWKV--PGTDEYTSRDPRPELYDSHYFLDNVLLALEQTSFCDFEVQFEVVHNALHSFLGGRGRYSMSTLDYSAFDPVFFLHHATVDRIWAIWQALQKFRGLPFDETDCAVNLMNIPLHPFD : 1520
NpH     : LYVIQVEHALRERGSGVSVPYWDWTKRFNHLPALMDESTFYNSRKNEMNTNPFHRGSI--SFINSHTSRDFQPELFNSDYLYENTLLALEQNDFCNFEVQLEVLHNAIHAWIGGRDTYSMSSLDYAAYDPIFFIHHSTLDRIWAIWQELQRYRGLPYNEANCALPLMNQHLRPFN : 1522
          L5V  VE AL1  GS V  6PYWDWT      LP 6     T551SR      NpFf    6         T3RDp    L5          61ALEQ  5C1FE  Q  E6  HNA  H   6GG4   yS6S  LD5  A5DP6Ff6hHa   DRI5AIWQ  LQ   R        5  E  1CA6n  M    6  PF1
```

```
KLH1   : KSD-NNDEATKTHATPHDGFEYQNSFGYAYDNLELNHYSIPQLDHMLQERKRHDRVFAGFLLHNIGTSADGHVFVCLPTGEH-TK-DCSHEAGMFSILGGQTEMSFVFDRLYKLDITKALKKNGVHLQG--DFDLEIEITAVNGSHLDSHVIHSPTILFEAGTDSAHTDDGHTEP : 1674
KLH2   : NPELNSDSMTLKHNLPQDSFDYQNRFRYQYDNLQFNHFSIQKLDQTIQARKQHDRVFAGFILHNIGTSAVVDIYICVEQGGE-Q--NCKTKAGSFTILGGETEMPFHFDRLYKFDITSALHKLGVPLDGH-GFDIKVDVRAVNGSHLDQHILNEPSLLFVPGERKNIYYDGLSQH : 1685
LsH1   : DDQLNHGDLTHRYSRPADTFDYRNHFHYEYDTLEFNHMTVPQLEALLKKRKESGRVFAGFLLHNIGLSAVANIYVCVPSGPR-GKKSCNHPAGSFSILGGELEMPFVFDRLYKYDITKTVRDLGLKLDNAANFELRVDIKAVNGSLLDSDILPKPTILFEPGTGERQKSSGEVTN : 1705
LsH2   : NDSLNHGDLTHRYSQPAETFDYRNHFHYEYDTLEFNHLTIPQLDNLLKSHQKSGRVFAGFLLHNIGLTAVVDIYVCVPSGAH-NKQSCNHKAGSFSILGGELEMPFAFDRLYKFDITRAVRDLGLKLDNAANFELKVDIRAVNGSLLDSSILPPPTIIFVPGTSERQEAHGAVSS : 1698
MtH400 : VKAQNEFDITHKYSRPVDVWDYSDHLQYHYDNLLLNDWTIPQLEEVLKTQRSRDRMFAGFLLHNIGTSADVELHICVATGNG-DR-NCNNPAGKFSILGGEYEMPFTFDRLYKYDISDSIRKLGLKLDSGANFDLKVQIHAYNGSYLDPSLLKEPTIIFEPGENTFQTQDGKTQR : 1708
NlH1   : NTDINLFRLTHEYSRPADVWDYSGHLNYHYDNLDFHHWSISELDEVLKKQRSRDRMFAGFMLHGIGTSAVVEINVCVDTGVG-GR-SCHHPAGKFCILGGEYEMPFTFDRLYKHDISDAIRKLGLKLDSAADFDLEVKIHSFNGSYIDASLLKKPTIIFQPGEGHTQDDEGHPIR : 1703
RtH1   : NKDINHFGLTYKHSRPADVWDYSRHLDYHYDNLQLHGWSIAQLDEVLKKQRSRDRMFAGFLLHNIGTSAVVEIDVCVDTGDG-GR-SCHHPAGKFSILGGEYEMPFTFDRLYKHDITDAIRKLGLRLDSAADFDLEVKIHSYNGSYIDASLLHPPSIIFQPGEGKTQDDEGHPIS : 1702
NlH2   : DKEMNDCRVTHKYSRPKDVFDYSHHFDYHYDTLEFNSWTIPQLEEVLKKQRSHDRLFAGFMLHSIGTSAVVEIDICVHTGAG-GL-SCDHPAGKFCILGGEFEMPWNFDRLYKYDITDTVRQLGLPLDSAADFEVHVKLHAYNGSYIDATPLGKPKILFQPGEGKSQDDHGHTHR : 1692
RtH2   : DKDLNDCWVTHKYSRPKDVFDYSHHFDYHYDTLEFNSWTIPQLEEVLRKQRSHDRLFAGFRLHNIGTSADVEIDICVKSGGG-AL-SCDHPAGKFSILGGEFEMPWIFDRLYKYDITDTVRKLGLPLDSAADFEVHIKLHAFNGSYIDATPLGHPTIMFEPGEGKSQDDQGHTHR : 1693
NpH    : NVSVNHDRNTLTLNKPNDAFDYQNHFHYRYDNLDFHGLSIPQLEHELHERQTHDRVFVGFLLHGIQASAYVRIYICVPTQRGSSKENCNNYAGIFSILGGVTEMPWHFDRLYYYEITHALSNLGLSHRSHFRIKTDINAVNGTHFDSHIFPDPTISFVPAPGSKNATHYQEEEHH : 1697
           N T P d 5dY h Y YD L 36 L 6 dR6FaGF LH Ig 3A v 6 6C6 g C AG F ILGG EMp5 FDRLYk dI3 6 lG6 ld f 6 ngs d p 6f pG g

KLH1   : VMIRKDITQLDKRQQLSLVKALESMKADHSSDGFQAIASFHALPPLCPSPAASKRFACCVHGMATFPQWHRLYTVQFQDSLRKHGAVVGLPYWDWTLPRSELPELLTVSTIHDPETGRDIPNPFIGSKIEFEGENVHTKRDINRDRLFQGSTKTHHNWFIEQALLALEQTNYCDF : 1849
KLH2   : NLVRKEVSSLTTLEKHFLRKALKNMQADDSPDGYQAIASFHALPPLCPSPSAAHRHACCLHGMATFPQWHRLYTVQFEDSLKRHGSIVGLPYWDWLKPQSALPDLVTQETYEHLFSHKTFPNPFLKANIEFEGEGVTTERDVDAEHLFAKGNLVYNNWFCNQALYALEQENYCDF : 1860
LsH1   : YLVRKEINSLSPREVQSLYSAMEALQADSSADGWQSIASFHAIPPLCPSPSAENRYACCLHGMATFPQWHRLYTVQVEDALKRHGALVGIPYWDWSRVSDSLPHFIDDENFVDPVTGDQKANPWKKARIEF--ENSETEREVVSDRLFKRGPHGWDTWLFNQALLALEQEDYCNF : 1878
LsH2   : FVVRKNVDALSQREALSLYNAMRALQADSSADGFQAIASFHALPPLCPSPAAINRYACCLHGMATFPQWHRLYTVQVEDALRRHGSLVGIPYWDWTRQSKSLPEFFSDPTYPDPDGGDPVDNPWYKANIEF--EHAVTERDVKEEFLFKVGKHGWDTWLFNQALYALEQEDYCDF : 1871
MtH400 : NMIRRNVLTLSLAERRSLVLAMRRLQEDHSADGFQALASFHALPELCPYPEAAKRYACCVHGMASFPQWHRLYTVQFEEALRRHGSLVGIPYWDTSRPQKALPAFATDEKFTDPVLNVEFDNPWLGADIEF--ENSHTEREPNLARLGEEGEHGYDTWLYEQYLLALEQDNYCDF : 1881
NlH1   : DLVRKSVWSLSPAEHRSLVLAMRSLQHDSSADGFQSLASFHALPPLCPYPEATERFACCIHGMATFPQWHRLYTVQFEDALRRHGPLVGIPYWDTVVPQSELPPFLHEATWDDPLFHANFTNPWHGADIEF--NHQHVARDFNMDQLAKKGPKGYDTWSWKQFVFALEQEDFCDF : 1876
RtH1   : DLVRKSVWTLSPAERRSLVLAMKSLQADSSADGFQSLASFHAQPPLCPYPEATKRFACCVHGMATFPAWHRLYTVQFEDALRRHGALVGIPYWDTVVPQSELPPFFNDEVWDDPLFHANFPNPWQGAEIEF--NHHKVARDFDMDQLAKKGPKGYDTWSWKQYIFALEQEDYCDF : 1875
NlH2   : NLVRKNVRNLSPAEHRSLVLAMKSLQADSSADGFQSLASFHAQPPLCPYPEASKRFACCIHGMATFPEWHRLYTVQFEDALRRHGSLVGIPYWDTVVPQDDLPALFNDEIWDDPLFHNNFTNPWHGADIEF--NHQHVARDIDMDKLSKKGPKGYDTWSWKQYIFALEQEGYCDF : 1865
RtH2   : NLVRKSVRNLSPAERRSLVLAMKSLQEDSSADGFQSLASFHAQPPLCPYPEANKRFACCVHGMATFPEWHRLYTVQFEDALRRHGSVVGIPYWDTVVPQEDLPAFFNDEIWDDPLFHANFTNPWNGADIDF--NHQKIARDINMDKLSKKGPKGYDTWSWKQYIYALEQEDYCDF : 1866
NpH    : HFVRKEVSRLSIYEIHEVVYAFRRMQRDMSADGFEYIASYHADPPLCPNPKALHRYACCIHGMATFLHWHRLYVVQFEQSLHRHGSDIGFPYWDWTRPITHIPALLKDVNYTDPFTNSVVGNPFNHGHLSFI-SPEVMTQRDVQDKLFKQPKLGRNTWLHDNVILALEQTDYCNF : 1871
           6R4 6 L 2 s6 A 6q D SaDG52 6AS5HA PpLCP P A R ACC6HGMA3Fp WHRLYtVQf2d L 4HG 6G PYWD p 6P dp NP5 a 6eF r L g g tW q 6 ALEQ 5C1F

KLH1   : EVQFEIMHNGVHTWVGGKEPYGIGHLHYASYDPLFYIHHSQTDRIWAIWQSLQRFRGLSGSEANCAVNLMKTPLKPFSFGAPYNLNDHTHDFSKPEDTFDYQ-KFGYIYDTLEFAGWSIRGIDHIVRNRQEHSRVFAGFLLEGFGTSATVDFQVCRTA-GDCEDAGYFTVLGGEK : 2022
KLH2   : EIQFEILHNGIHSWVGGSKTHSIGHLHYASYDPLFYIHHSQTDRIWAIWQALQEHRGLSGKEAHCALEQMKDPLKPFSFGSPYNLNKRTQEFSKPEDTFDYH-RFGYEYDSLEFVGMSVSSLHNYIKQQQEADRVFAGFLLKGFGQSASVSFDICRPD-QSCQEAGYFSVLGGSS : 2033
LsH1   : EIQFEITHNAIHSWVGGSKEHSLGHLHYASYDPAFFVHHSNTDRLWAVWQALQKYRGYDPNEANCALEQMKDPLKPFSFGPPYNLNPLTKEHSRPEDTFNYADHFHYRYDNLEFVGLSIPQLDAFIKERHEHDRVFAGFLLKGFKKSALVDFDICAN--GNCFPGGYFTVLGGSA : 2051
LsH2   : EVQFEITHNAIHSWLGGSKNYSLGHLHYASYDPAFFIHHSNTDRLWAVWQALQKYRGHDPNEANCALELMREPLKPFSFGPPYNLNQMTQDNSRPEDTFNYEDHFHYRYDDLQFVGMSIPRLDAFIKEKQEHDRVFAGFLLKGFGQSALVSFEICNDDMSNCFEGGYFTVLGGSQ : 2046
MtH400 : EVQFEIAHNAIHAWVGGSKIHSMGHLHYASYDPIFLLHHSSTDRIFAVWQELQRLRGKDPNEANCALEIMHEPLKPFSFGSPYNLNENTQRYSRPEDIFDYQSHFHYEYDSLEVLGMSPAQLEDAIHKRQEKDRVFAGFLLKGIGTSAHVEFTICNEA-GVCTTAGDFDILGGSA : 2055
NlH1   : EVQFEIAHNALHAWVGGHEEYSMGHLHFASYDPVFILHHSNMDRILALWQELQAFRGHDPYNINCALELMREPLKPFSFGPPYNLNSNTREHSKPEEALDYHTHFHYQYDSLELQGMNVQRIQDYINKQKEKARVFAGFLLEGFGSSAHVAFSVCKEG-GECTDAGFFDVLGGQL : 2050
RtH1   : EVQFEIAHNAIHAWVGGKEEYSMGHLHYASYDPIFILHHSNTDRIFALWQKLQKFRGHDPNNVNCALELMREPLKPFSFGSPYNLNDNTREHSKPEETLDYHTHFHYDYDSLELQGMDVHRLQDYINQQKEEDRVFAGFLLQGIGTSAHVAFSVCKDG-GECTSAGFFDVLGGAL : 2049
NlH2   : EVQFEIAHNAIHAWIGGDEEYSMGHLHYASYDPVFILHHSNIDRIFALWQELQEFRGHDPNEVNCALEMMREPLKPFSFGAPYNLNPITEEHSKPEDSFDYKGHFHYEYDQMELQGMTAERLQDYINKQKERDRVFAGFLLGGIETSALVSFDVCKPD-GDCTHAGDFDILGGSL : 2039
RtH2   : EVQFEIAHNAIHAWVGGHEEYSMGHLHYASYDPVFILHHSNTDRIFALWQELQKFRGHDPNEVNCALEMMREPLKPFSFGAPYNLNPTTKEHSKPEDTFDYKGHFHYEYDHLELQGMNVQRLHDYINQQKERDRVFVGFLLEGIGTSAHLDFSICKPD-GECTHAGYFDVLGGSL : 2040
NpH    : EVQFEIVHNAIHAWVGGSQEHSLSHLHFAAYDPAFYLHHSNVDRIWVIWQELQKIRGLSAYEANCALELMNEPLKPFAFGSPYNLNEMTRKYSLPEDTFNYKDHFDYEYDMLEFSGMTIPQLEDYIKESLHHERIFAGFLLHGIGQSANVDFEVCDNQ-NHCEDAGYFSILGGSL : 2045
         E6QFEI HNa6H W6GG s6gHLH5AsYDP F 6HHS DR6 a6WQ LQ RG e nCA6e M PLKPFsFG PYNLN T S PEd f1Y hF Y YD 62 G 6 6 e R6FaGFLL G g SA 6 F 6C C aG F 6LGG

KLH1   : EMPWAFDRLYKYDITETLDKMNLRHDEIFQIEVTITSYDGTVLDSGLIPTPSIIYDPAHHDISSHHLSLNKVRHDLSTLSERDIGSLKYALSSLQADTSADGFAAIASFHGLPAKCNDSHNNEVACCIHGMPTFPHWHRLYTLQFEQALRRHGSSVAVPYWDWTKPIHNIPHLFT : 2197
KLH2   : EMPWQFDRLYKYDITKTLKDMKLRYDDTFTIKVHIKDIAGAELDSDLIPTPSVLLEEGKHGINVRHVGRNRIRMELSELTERDLASLKSAMRSLQADDGVNGYQAIASFHGLPASCHDDEGHEIACCIHGMPVFPHWHRLYTLQMDMALLSHGSAVAIPYWDWTKPISKLPDLFT : 2208
LsH1   : EMPWQFDRLFKYEITDQLNEHKLRFDDEYTFQIKIRAPDGTELSSDLIGTPSVILEPFKRDYDLEKVTPNFVRHDLSTLTERDLQGLKSALRDLQLDESADGWASLASFHGAPSMCENESGAKVACCIHGMPTFPHWHRLFTVQVERALQRHGSAVAIPYWDWTKPITALPEIFT : 2226
LsH2   : EMPWRFDRLYKYEITEQLEEHNLRYDDNYHFHVHVKNIDGTMLPGKLIPDPTVILVPAQSDHDLDNVTPNLIRRDLSTLSERDIQSLQAALRDLQLDQSNDGWASLASFHGAPNRCPDPANPTVACCQHGMPTFPHWHRLFTVQIEQALQRHGSAIAIPYWDWTKPIDELPKIFT : 2221
MtH400 : EMPWSFDRAYKYDITNVLEKKGLEATDAFKVKVAITAQDGTALDSSLLPEPSIILQHATRDQGQYELAPNRVRRSLSDLTERDVMSLKSALHDLQEDDSATGWQSLAAYHGVPALCPSPEEAKYACCIHGMPTFPHWHRLYVLAVEHSLIKHGSSVAIPYWDWTNPLDKLPELFT : 2230
NlH1   : EMHWKFDRLYKYEITDVLAAKGLEVHDSFHIEVTITAPNSTVLPSTLIPTPSIIYVPKSHDTELHEVAPNRVRRSLADLTERDAKNLKSAIRDLQKDDSKDGYQHIAGFHGAPALCPTPEAAEYACCIHGMPTFPHWHRLYAVEMEDALIRHGSGVALPYWDWTLPITELPHLFT : 2225
RtH1   : ETPWRFDRLYKYEISDVLKEKGLEVRDAFHIELTITASNGTALPSSLIPEPSVIYAPKSHDTELHEVAPNRVRHDLADLTDRDIKNLKSAIRDLQNDISKDGYQHIATFHGAPALCPDPNAPTHACCLHGNPTFPHWHRLYAVEMEDALIRHGSGVALPYWDWMKPITELPELFT : 2224
NlH2   : EMPWQFDRLYKYEITDVLAHKGLDVHDVFDIKVTITGPHSEDISSDKLPHPTVLFVPKSRDIEQHEVAPNRVRHELGDLSDRDVQNLKAAIRDLQLDDSRDGYQNIASFHGSPALCPSPDAPEYACCNHGNPTFPHWHRLYAVEMEDALIRHGSGVALPYWDWTYPLTKLPDLFT : 2214
RtH2   : ETPWQFDRLYKYEITDVLESKGLDVHDVFDIKITSTSWDNEDISTDRFPPPSVIYVPKSRDIELHEVAPNRVRNDLSKLSERDIQNLKSAIRDLQLDKTIDGYQNIASFHGAPALCPSAENAEYACCIHGVPTFPHWHRLYTVEMEDALIRHGSGVALPYWDWSVAITDLPDLFT : 2215
NpH    : EMPWAFNRLYRYDITHTLETMHLNYDDNFHINIHIHDMAGNDLDSHLLQKATILRQPAAEHHEVHH-PLNRIRHNINDLSERDIQSLKSALIKVKEDHSNFGFQTITSYHGLPASCPTEEATYACCMHGMAAFPHWHRLYALQFEESLLRHGSSVALPYWDWTHKITGLPHLLS : 2219
         EmpW F1R154Y I3 L L d 5 6 6 l p p366 p d N 6R 6 L3eRD Lk A6 6q D G5 6a 5HG Pa C ACC HG ptFPHWHRL5 6 e aL HGS 6A6PYWDW p6 6P 6f3

KLH1   : DKEYYDVWRNKVMPNPFARGYVPSHDTYTVRDVQEGLFHLTSTGEHSALLNQALLALEQHDYCDFAVQFEVMHNTIHYLVGGPQVYSLSSLHYASYDPIFFIHHSFVDKVWAVWQALQEKRGLPSDRADCAVSLMTQNMRPFHYE-INHNQFTKKHAVPNDVFKYELLGYRYDNL : 2371
KLH2   : SPEYYDPWRDAVVNNPFAKGYIKSEDAYTVRDPQDILYHLQDETGTSVLLDQTLLALEQTDFCDFEVQFEVVHNAIHYLVGGRQVYALSSQHYASYDPAFFIHHSFVDKIWAVWQALQKKRKRPYHKADCALNMMTKPMRPFAHD-FNHNGFTKMHAVPNTLFDFQDLFYTYDNL : 2382
LsH1   : KEDFYDAWRDEVVDNPFARGFIPTENTYTVRDIRPELFQTSKDGSHSDLFELVLSALEQTDYCDFEVQFEVMHNAIHYLVGGLQTYSLSSLEYSAYDPIFFIHHSFVDKIWVVWQELQQRRHLPSNTANCALNYLAEPMKPFNLD-LNINAFTKKHAVPRDVFNHEELGYHYDNL : 2400
LsH2   : DEDFYDVWRDEVVANPFARGYVPSEKVYTVRETKSELYDKGRGGKNSALIYYVISALEQTDYCDFEVQFEVIHNSIHYLVGGEQTYSLSSLEYSAYDPIFFIHHSFTDKIWAVWQELQKRRRLPYLKANCALNYMAEPMKPFSIDGLNLNKFTKEHAVPDKLFDYEELGYAYDTL : 2396
MtH400 : KQTYYDAWRDEVYDNPFARGYVKGDEAYTVRDPQPELKERSRDGKHSVLFDQVLLALEQTDYCDFEVQYEVTHNAIHYLVGGPQTFSLSSLHYSSYDPIFFVHHSFVDKIWAVWQALQRKRGKPSERADCAVNYMYDDMHPFDSAKLDPDPTIRSHANPSTVFNIYDLGYKYDDY : 2405
NlH1   : SESYYDPWRDEVVPNPFVRAYIKVAGGYTVRDPQPELHKLSKDGNHSILFDEILLALEQTDYCDFEVQYEVTHNAIHYLVGGRQVYSLGSLDYSSYDPIFFVHHAFMDKVWAVWQEMQKRRGLVYDRADCAVNYMKHKMHPFDWDDLNPDVRTREHAMPQSVFDYEDLGYHYDNF : 2400
RtH1   : SESYYDPWRDEVVPNPFVRAYIKAANGYTVRDPRPALKKLSHDGKHSILFDEVLLALEQTDYCDFEVQFEVTHNAIHYLVGGRHLYSLSSLEYSSYDPIFFVHHSFVDKIWAVWQELQKRRGLTYDRADCAVNFMQHKMHPFDWDDLNPDVRTREHAHPQTVFDYEDLGYHYDDF : 2399
NlH2   : SESYYDAWRDEVIPNPFVRAYIDVAKGYTVRNVQPELTKLSKDGKHSVLFDELLLVLEQTDYCHFEVQYEVLHNAIHYLVGGRQVYSLATLDYASYDPIFFVHHSFVDKVWAVWQELQKRRGMVYDRADCAVNYMNHPMHPFDWESLNPDVRTREHASPQTVFDYEDLGYHYDNF : 2389
RtH2   : SESYYDAWRDEVIPNPFARSYIDVAKGFTVRDPQPELTKLAKDGKHSMLFDELLLALEQTDYCDFEVQFEVLHNAIHFLVGGRQEYSLSSLEYSSYDPIFFVHHSFVDKVWAVWQELQKKRGLVYDRVDCAVNYINQPMHPFDWENLNPDVRTREHASPSSVFDYEDLGYHYDNF : 2390
NpH    : NPDYYDALHKQVTENPFLRGHIEVAETYTVRNTQPELFEPSKDGKHSLLYDEILLALEQRDYCDFEVQFEVVHNAIHYLVGGHQRYSMSSLEYASYDPLFFIHHSNVDRLWAIWQALQQYRRIPYNKAYCALEQMTHAMKPFDRE-TNPNKNTHDHAIPNSVFNYDDLGYSYDNL : 2393
            5YD wr V NPF 4 6 5TVR L g hS L 6laLEQtD5CdFeVQ5EV HNaIH5LVGG q 5s6s3l Y sYDP FF6HHsf D46Wa6WQ 6Q R a CA6n 6 M PF 1 1 t HA P 6F LgY YD
```

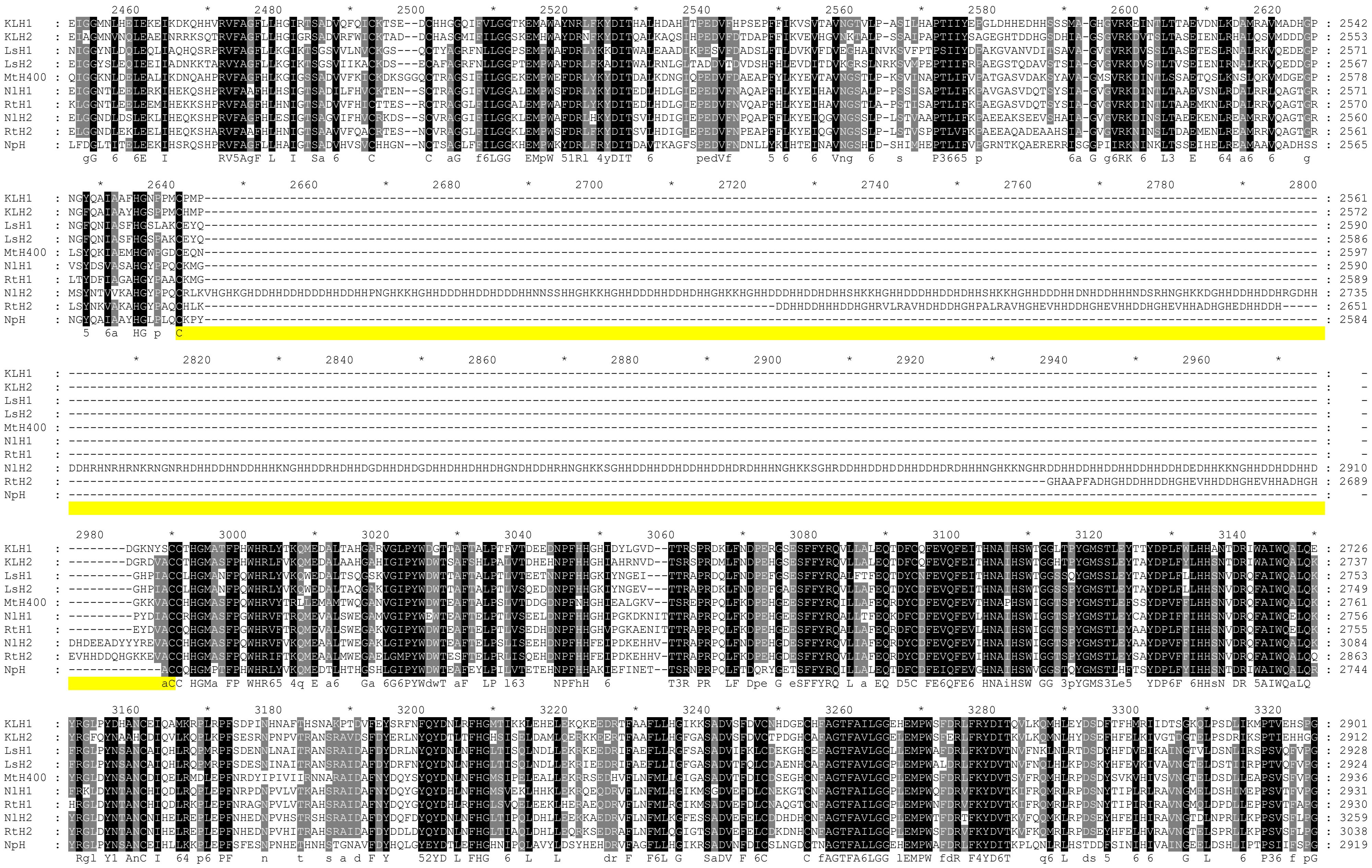
KLH1
KLH2
LsH1
LsH2
MtH400
NlH1
RtH1
NlH2
RtH2
NpH

```
                *        3340         *        3360         *        3380         *        3400         *        3420         *        3440         *        3460         *        3480         *        3500
KLH1   : GKHHEKHHEDHHE-----------DILVRKNIHSLSHHEAEELRDALYKLQNDESHGGYEHIAGFHGYPNLCPEKGDEKYPCCVHGMSIFPHWHRLHTIQFERALKKHGSHLGIPYWDWTQTISSLPTFFADSGNNNPFFKYHIRSINQDTVRDVNEAIFQQTK--FGEFSSIFY : 3063
KLH2   : DHHGGDTSGHDHSERH--------DGFFRKEVGSLSLDEANDLKNALYKLQNDQGPNGYESIAGYHGYPFLCPEHGEDQYACCVHGMPVFPHWHRLHTIQFERALKEHGSHLGIPYWDWTKSMIALPAFFADSSNSNPFYKYHIMKAGHDTARSPSDLLFNQPQ--LHGYDYLYY : 3077
LsH1   : VKRFYEKVAEKTVVRD--------DKLVRKNVNQLTLDEAANLRNALNQLQNDQGPNGFEAIAGFHGAPFRCPATGDDKYACCVHGMPVFPHWHRLFTVQIEQALKSNGALIGVPYWDWTTPVKSLPSFFGDDADHNPFASFRIAAANEKTTRQVQSELYSERK--VHGFPYLFY : 3093
LsH2   : VKRYYEKLAERTVHRE--------DKLTRKDVNTLTLAEATNLRNALSQLQQDAGLNGFEAIAGFHGAPFKCPATGTDKYACCVHGMPIFPHWHRLLTVQFEQALKAHGASVGVPYWDWTAPVRSLPSLFGDSADFNPFYSYFISFVGKKTTRDVKDELFNPPT--INGFNQLYY : 3089
MtH400 : KRGRKAREDYRPTVS---------EDLIRKEVSSLSLEEVSNLKNALYKLQNDHGPNGFEAIASFHGDPGLCPEGSGSKYACCQHGMPVFPHWHRLLTVQFERALKAKGSVVGVPYWDWTRPSRGIPQLFSNTYGNNPFLTYEIQAASAFVERDVQVDALEHTTGHEGFEDSLFH : 3102
NlH1   : RKVGSTKAVVHEDPMPTET-----EDLTRYEISALSLEQISNLRNALYKLQNDFGPNGFEAIASFHGAPGVCPQNASKHHSCCQHGMPAFPHWHRLLTVQFEHALKEKGAGVGVPYWDWTRPSKGLPSFFTDPSNINPFKTYLMSFNDHHIERQVHEDLFSHSS--EADPESLFH : 3099
RtH1   : KKSGSGRAVVYEEPTPSET-----QDLTRHDLSSLSMAQISNLEDALYKLQNDHGPNGFEAIASYHGAPGLCPENATEHYSCCQHGMPAFPHWHRLLTVQFEHALKEKGAMVGVPYWDWTRPAKGMPTLFTDNSAINPFYTYLMSFNNHHIERKVHEDLFSHSA--EGDPESLFH : 3098
NlH2   : RKTYSEVAEDHDEHTPTES-----DDMVRYEVSDLSLAQVSNLRDALYKLMNDHGPNGFESIASFHGAPGLCPENATEHYACCQHGMSAFPHWHTLLTVQFEHALHDKGALVGIPYWDWTRPSKSLPSLFTDPSEGNSFRTYKISFNNQYIQREVQDELFNHPS--EGDPESLFH : 3427
RtH2   : RKTRGQAADAHHEEQPTNTTTDSDDDLVRYEVSAMSLAQVNNLRTALNKLMHDSGPNGYEAIAGFHGAPGLCPEKAEEHYSCCQHGMPAFPHWHSLLTVQFEHALREHGSLVGVPYWDWTRPSKALPSLFTDPSESNPFKTYKISFKGQYIKREVHEDLFNHPS--EGDPESLFH : 3211
NpH    : RGAQH------------------------------------------------------------------------------------------------------------------------------------------------------------------------ : 2924
                                  r             l  al  l  d g ng e ia  hg p  cp      y cc hgm  fphwh l t qfe al   g   g pywdwt      p  f      npf            r v

                     *        3520         *        3540         *        3560         *        3580         *        3600         *        3620         *        3640         *        3660         *
KLH1   : LALQALEEDNYCDFEVQYEILHNEVHALIGGAEKYSMSTLEYSAFDPYFMIHHASLDKIWIIWQELQKRRVKPAHAGSCAGDIMHVPLHPFNYESVNNDDFTRENSLPNAVVDSHRFNYKYDNLNLHGHNIEELEEVLRSLRLKSRVFAGFVLSGIRTTAVVKVYIKSGTDSDDE : 3238
KLH2   : LALSTLEEDNYCDFEVHYEILHNAVHLWLGGTETYSMSSLAFSAYDPVFMILHSGLDRLWIIWQELQKLRKKPYNAAKCAGHMMDEPLHPFNYESANHDSFTRANAKPSTVFDSHKFNYHYDNPDVRGNSIQEISAIIHDLRNQPRVFAGFVLSGIYTSANVKIYLVREGH-DDE : 3251
LsH1   : LALTTLEEDNYCDFEVQFEVLHNEIHADIGGSGTYSMATLDYSAFDPFFMIHHSSIDRIWVIWQELQKLRHKPFNGASCAGHIMERPLQPFSYPEVNKNEFTRLNSVPNVVFDSERLGYKYDKLELNGNSVEEINNIIKNLHHQERIFVGILAFGHQKSLTIHISLINDND-EAF : 3267
LsH2   : LALSTLEEDNYCDFEVQYEILHNQIHALIGGTGTYSMATLDYSAFDPFFMIHHSSIDRIWVIWQELQKIRKKPFNAAHCGGHLMETPLQPFSYPEVNPNDLTRLNAVPNLVFDSERFGYEFDKLELNGHDVSELNGIIQRLRGNTRYFLGFVLYGVRQTVGVEVTLISN-K-KRY : 3262
MtH400 : QALETLEETSYCEFEVQFEMLHNAVHALVGGTKTHSMATLEWSAFDPFFMVHHSSIDRIWRIWQELQRLRKKPFNVARCAMRYLRKPLEPFSYASVNTDEVARTNSRPIDIFDTAKFHYDFDNLDLGGHSIGEVNKMINDMRSKTRLFVGFVLSGIETSATVKIEMDDSQG-NSH : 3276
NlH1   : QALEMLEETNYCDFEVHYEMLHNAVHELIGGNHTYSMSTLEYSAFDPFFMVHHSSIDRIWAIWQTLQKLRHKPFNYAVCAARSMNRPLEPFSYDTINKDSLTHDNAMPSQIFDSHKFHYHYDNLQLNGHSVTELYKMIHSMQAQTRTFAGFVLSGFGSSARVHVDIVKG-A-DTA : 3272
RtH1   : QALELLEETNYCDFEVQYEMLHNAVHELVGGPHKYSMSTLEYSAFDPFFMVHHSSIDRIWQIWQTLQKLRHKPFNYAVCAARSMYKPLEPFSYESINPDPLTRENSKPAQIFDTHKFHYHYDNLNLNGHSVAELNTMIHAMQAQSRTFAGFVLSGISTSARVHVDIYKG-D-EVA : 3271
NlH2   : QALELLEETSYCDFEVQYEMLHNAVHELVGGRHTHGMSTLEFSAFDPLFMVHHASIDRVWKIWQELQKLRHKPFNYAVCAHRNLYRHLEPFSYETVNTDALTRNNAQPVQIFDKTKFHYHYDNLELNGHDLHELEEMIHEMQTHTRVFAGFVLYGFGTSARVHVDIMKD-G-EEV : 3600
RtH2   : QALELLEQTSYCDFEVQYEMLHNAVHELVGGPNTYSMSTLDYSAFDPFFMVHHASIDRLWLIWQQLQKLRGKPFNYAVCAARLLYRPLEPFSYESVNADPLTRNNAKPVQIFDREKFHYRYDSLELSGHALQELEDMIHDMQTHTRLFAGFVLHGFGTSARVHVDIVKD-G-HVE : 3384
NpH    : ---------------------------------------------------------------------------------------------------------------------------------------------------------------------------- :    -
           al  le   ycdfev  e lhn  h   gg    sm  l  sa dp fm hh s d  w iwq lq  r kp n a ca       pl pf y   n    tr n  p   fd   f y  d l   g    e            r f gf l g

          3680         *        3700         *        3720         *        3740         *        3760         *        3780         *        3800         *        3820         *        3840
KLH1   : YAGSFVILGGAKEMPWAYERLYRFDITETVHNLNLTDDHVKFRFDLKKYDHTELDASVLPAPIIVRRPNNAVFDIIEIPIGKDVNLPPKVVVKRGTKIMFMSVDEAVTTPMLNLGSYTAMFKCKVPPFSFHAFELGKMYSVESGDYFMTASTTELCNDNNLRIHVHVDDE : 3408
KLH2   : NVGSFVVLGGPKEMPWAYERIFKYDITEVANRLNMHHDDTFNFRLEVQSYTGEMVTHHLPEPLIIYRPAKQEYDVLVIPLGSGHKLPPKVIVKRGTRIMFHPVDDTVNRPVVDLGSHTALYNCVVPPFTYNGYELDHAYSLRDGHYYIAGPTKDLCTSGNVRIHIHIEDE : 3421
LsH1   : DGGNIHILGGEKEMPWAYERIMKLDVTEAIRKGKISTDHAVKARFTSTDYQGNLNHQDTDYAIIVERHAEQDYDVVEIPVGRKYPLPPKIVLKKGSRVKFYPGSDTFNSPLENLGSYTSFSKCSIPPFSYQSYALGVVHTLQPGDYFFVPKDKALCEAGK-RIQITVEDE : 3436
LsH2   : PAGNFYVLGGEKEMPWAYERIAKYDVTDVFHKAKLSDDKPVVGEFYSSLYNGQANNNVTEEVIVVEKPSDVNYDIVIIPISTGNIIPPKIILKKGSRVKFVSSNISV--PMEELGSYTNLQKCKVPPFSFHSYPFGVVHTLAPGDYFFVPKSVQLCEAGR-RIQITVEDE : 3429
MtH400 : EVGTFYVLGGHNEMPWAYERVYKYEITEAAKKYGLDHDSVFNFHHKVVKYDGQELSAQFKAPILVERPAGVDYDVAIFQLKKDNDLAPKVVLPRGTRIRFHSAEDGVSSVLREMGSYTNAVYCSIPPGDANTYKLEVEYTLEPGDYFFVSNDEEKCKQGD-RIQISIDEE : 3445
NlH1   : SVGNFFVLGGPTELPWAYERIYKLDMTEATKKLGLAGSTSFEFKLTVTKYDGTPMDVHFPDPVILKRRANSHFDELILPIRKENKLPPKIVVRRGTRVLFHPTEVSVVGPIRELGSFTNSEHCAIPPGHAHSYELDVLHVLEPGDYYFVSNNLDACKAGS-RVQISVDEE : 3441
RtH1   : SVGNFFVLGGPSEMPWAYERIYKLDMTEAAKKLGLSGSSHFDFKLTVTKYDGSALDVKFPDPVIVKRRTNAEFDELILPIRKENKLPPKIVVRRGTRVLFHPTEEGILGPIRELGSFTNSKLCAIPPGHAHAYELDRLHVLEPGDYYFVSNNVESCKAGS-RIQISVDEE : 3440
NlH2   : KVGNFFVLGGPTEMPWAYERIYKLDMTDAVHELNQTENCDFDFQLTVTKYDGTQLDVVFPEPVIIKRRPNVESDELILPLRMVNRLPPKIVVRRGTRVIFHPLEDEVKGPLRELGSYTSSLHCTIPPGEAHAYDFDVPHDLEPGDYYFTVNDESQCEGGA-RFQITVDEE : 3769
RtH2   : KVGNFFVLGGASEMPWAYERIYKLDMTEAAEHLNMSDSCAFHFQLTVTKYDGTALDVEFPEPVVIRKRANTEFEEVILPLKTKNNLPPKIVVRRGTHVIFHAVDQALHGPIRELGSYTNSVHCAIPPGEAHAYAFDESHELEPGDYYFTANDAQQCHGGA-RIQLTVDEE : 3553
NpH    : ------------------------------------------------------------------------------------------------------------------------------------------------------------------------- :    -
            g f  lgg  e pwayer    d t                               y g                     d    p        ppk     g    f         p    gs t    c  pp                gdy         c  g  r       e
```

Conservation Shading:

100% Similarity Conservation - black

≥ 80% Similarity Conservation - grey
