# Supplementary material for: Hemocyanins of Muricidae: New ‘Insights’ Unravel an Additional Highly Hydrophilic 800 kDa Mass Within the Molecule
Source: J Mol Evol. 2021 Jan 13;89(1):62–72. doi: 10.1007/s00239-020-09986-6 (PMC7884596; doi:10.1007/s00239-020-09986-6)
Supplement: Supplementary file 3 — Electronic supplementary material 3 (PDF 697 kb) [file 239_2020_9986_MOESM3_ESM.pdf]

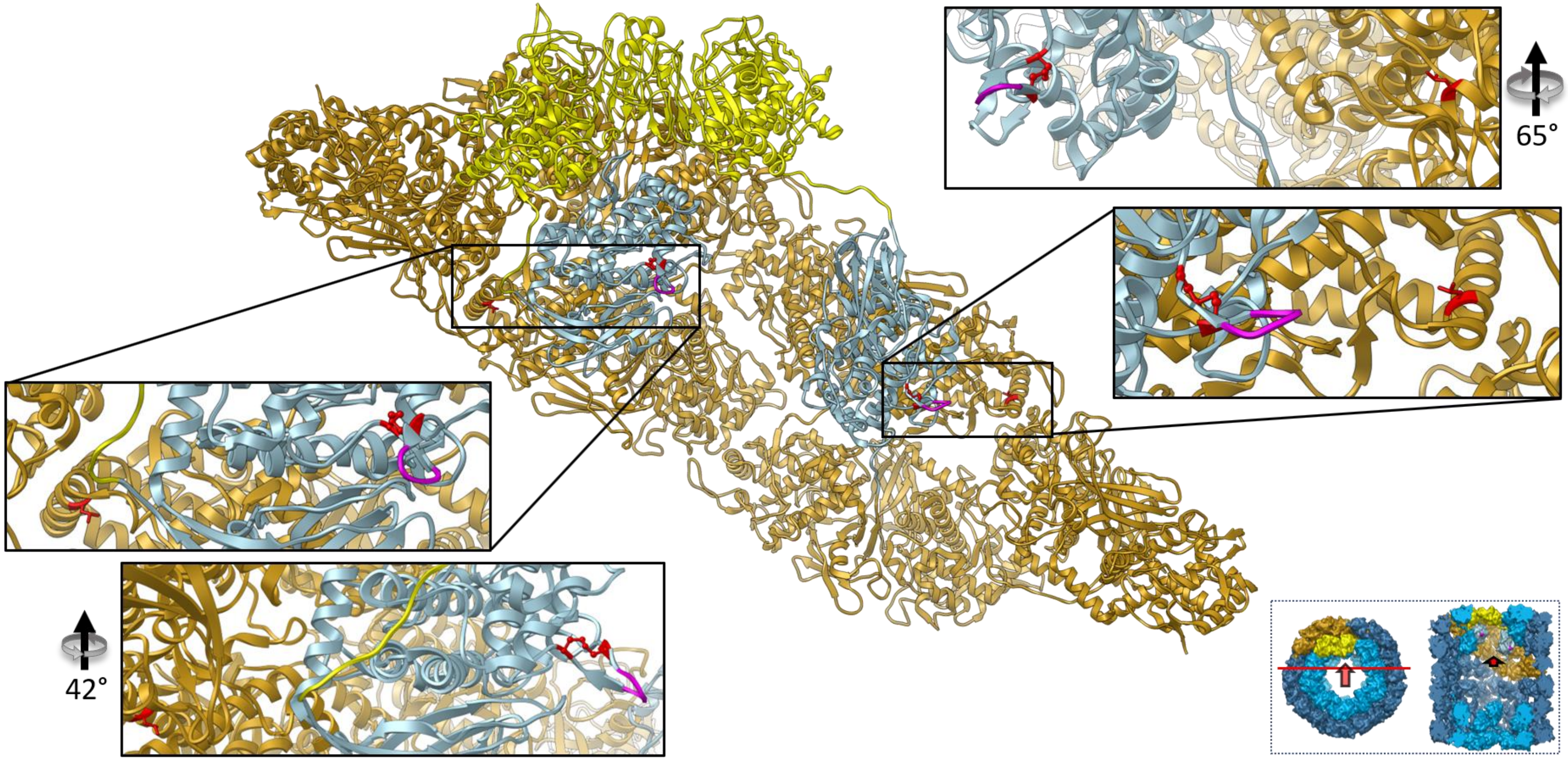

Supplement 3: Ribbon diagram of hemocyanin dimer. Shown is the model of a KLH1 dimer based on a 9 Å cryoEM structure (Gatsogiannis and Markl 2009, PDB: 4BED). Color coding: golden – wall (FU-a – FU-f); silver – FU-g; yellow – FU-h. The loop marked in pink is conserved in hemocyanins over all molluscan classes that have been analyzed so far and typically covers four to seven amino acids (five in KLH1). It is stabilized by a disulfide bridge marked in red and can be seen more detailed in the magnifications in the subwindows (rotated views shown next to rotating arrows). In addition, the leucine of the FU-d in KLH which is substituted by a cysteine in NlH and RtH is marked in red. It may be able to form a disulfide bridge with a second cysteine (presumably of FU-g; see hypothesis in discussion section). The smaller density models, shown on the bottom right, illustrate the lines of sight (red arrows) and the cutting surface (red line).
